# The α-Gal Epitope (Galα1-3Galβ1-4GlcNAc) as Therapeutic Agent in Cancer Immunotherapy, Vaccine Effectiveness Amplification and Injured Tissue Regeneration

**DOI:** 10.3390/ijms27062737

**Published:** 2026-03-17

**Authors:** Uri Galili

**Affiliations:** Department of Medicine, Rush University Medical Center, Chicago, IL 60612, USA; uri.galili@rcn.com

**Keywords:** anti-Gal antibody, α-gal epitopes, α-gal nanoparticles, cancer immunotherapy, oncolytic virus, viral vaccine, tissue regeneration

## Abstract

The α-gal epitope is synthesized in non-primate mammals and New-World monkeys by the glycosylation enzyme α1,3galactosyltransferase (α1,3GT), encoded by the *GGTA1* gene. Ancestral Old-World monkeys and apes synthesizing α-gal epitopes underwent extinction 20–30 million years ago. Their mutated offspring, with the inactivated *GGTA1* gene, survived and produced the natural anti-Gal antibody, specifically binding α-gal epitopes. Anti-Gal protected the surviving offspring from lethal viruses presenting α-gal epitopes, which killed α-gal-synthesizing parental primates. Anti-Gal constitutes ~1% of human immunoglobulins and is also produced in Old-World monkeys and apes. α-Gal epitopes can serve as therapeutic agents in several clinical disciplines: 1. *Cancer immunotherapy*: Engineering cancer cells to express α-gal epitopes results in anti-Gal binding to these cells and localized activation of the complement system that kills these cancer cells and recruits the antigen-presenting cells (APCs) dendritic cells and macrophages. Anti-Gal bound to cancer cells targets them for robust uptake by APCs, which process internalized tumor antigens (TAs) and transport them to lymph nodes for activation of cytotoxic T-cells. These T-cells kill TA-presenting metastatic tumor cells. Clinical trials demonstrated that such engineering is achieved by intra-tumoral injection of α-gal glycolipids, the use of recombinant α1,3GT, or the use of oncolytic viruses containing the *GGTA1* gene. 2. *Viral vaccines*: Inactivated whole-virus vaccines presenting α-gal epitopes bind anti-Gal, which targets them for extensive uptake by APCs, thereby increasing their immunogenicity by ~100-fold. 3. *Injured-tissue regeneration*: Anti-Gal binding to α-gal-presenting nanoparticles administered to wounds, into the post-myocardial infarction (MI) injured myocardium and into injured spinal cord, activates the complement system that recruits pro-regenerative macrophages, which orchestrate regeneration by recruiting stem cells and the secretion of pro-regenerative cytokines. All these findings suggest that α-gal/anti-Gal antibody interaction can serve as a novel therapeutic approach, applicable to various clinical settings.

## 1. Introduction: α-Gal Epitope and Natural Anti-Gal Antibody Distribution and Evolution

**Figure 1:**
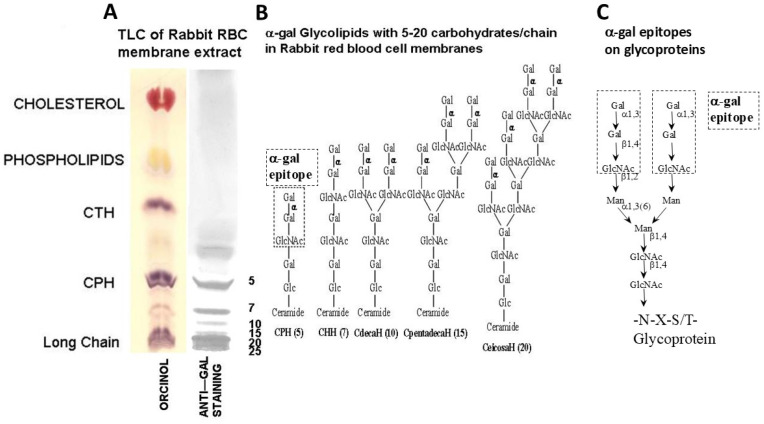
Natural glycolipids and glycoproteins presenting α-gal epitopes. (**A**) Glycolipids, phospholipids and cholesterol extracted from rabbit red cell membranes, separated on TLC plates and stained by orcinol (non-specific staining) or with anti-Gal bindings specifically to α-gal epitopes. CTH (ceramide tri-hexoside Galα1-4Galβ1-4Glc-ceramide) lacks the α-gal epitope and does not bind anti-Gal. (**B**) CPH (ceramide penta-hexoside) and glycolipids with 7, 10, 15 and 20 carbohydrates (hexosides) all have α-gal epitopes and bind anti-Gal. (**C**) α-Gal epitopes on N (asparagine)-linked carbohydrate chains of proteins. Adapted from Ref. [16] with permission.

The carbohydrate antigen “α-gal epitope” (also referred to as α-galactosyl and Galα1-3Gal) with the structure Galα1-3Galβ1-4GlcNAc-R is unique in that it is found on glycoproteins, glycolipids (Figure 1) and proteoglycans of many mammals but not in other vertebrates [1,2]. The α-gal epitope is synthesized in non-primate mammals, lemurs (prosimians evolved in Madagascar) and New-World monkeys (monkeys of South America) and is absent in Old-World monkeys (monkeys of Asia and Africa), apes (gibbon, orangutan, gorilla and chimpanzee) and humans [1,2]. The synthesis of α-gal epitopes is catalyzed by the glycosylation enzyme α1,3galactosyltransferase (α1,3GT) [3,4,5,6] in the following reaction:

$$\begin{array}{l} \;\;\;\;\;\;\;\;\;\;\;\mathrm{\alpha 1,3GT}\\ \mathrm{Gal\beta 1-4GlcNAc-R + UDP-Gal}\;\to \;\mathrm{Gal\alpha 1-3Gal\beta 1-4GlcNAc-R + UDP}\\ \mathrm{N-Acetyllactoamine}\;\;\;\mathrm{Uridine-}\;\;\;\;\;\mathrm{\alpha -gal epitope}\;\;\;\;\;\mathrm{Uridine-}\\ \;\;\;\;\;\;\;\;\;\mathrm{diphosphate}\;\;\;\;\;\;\;\;\;\;\;\mathrm{diphosphate}\\ \;\;\;\;\;\;\;\;\;\mathrm{galactose} \end{array}$$

The α-gal epitope was found to be a carbohydrate antigen of interest because as many as 1% of human immunoglobulins are the natural anti-Gal antibody [7,8,9,10] which specifically binds this epitope [11,12,13]. This binding can be demonstrated by thin layer chromatography (TLC) immunostaining of various types of glycolipids in mammalian cells, such as rabbit red cells (Figure 1A). Human natural antibodies are continuously produced throughout life without intended vaccination. The most well-known natural anti-carbohydrate antibodies are anti-blood group A and B antibodies. In contrast to these two natural anti-carbohydrate antibodies which are found in parts of human populations, based on their blood type, anti-Gal is produced in all humans who are not severely immuno-compromised [7]. Most anti-Gal production is in response to antigenic stimulation by carbohydrates of the gastrointestinal bacteria [14,15].

Anti-Gal comprises many antibody clones that bind to various facets of the α-gal epitope. In individuals of blood types A and O, anti-Gal also constitutes >85% of the anti-blood group B antibody clones, i.e., part of the anti-Gal antibody clones can bind both to the α-gal epitope and to blood group B (Galα1-3[Fucα1-2]Galβ1-4GlcNAc). This binding occurs because the fucose linked to the penultimate galactose does not interfere with binding of these anti-Gal antibody clones to the B antigen [17,18]. Such anti-Gal clones are absent in individuals with blood type B or AB, because of immune tolerance to self-carbohydrate antigens.

α1,3GT is encoded by the *GGTA1* gene [19,20]. The activity of α1,3GT in all non-primate mammals tested, in lemurs and in New-World monkeys, vs. its inactivity and the corresponding lack of α-gal epitopes in Old-World monkeys, apes and humans (Old-World primates) implies that the *GGTA1* gene was inactivated in ancestral Old-World primates (monkeys and apes) ~20–30 million years ago, after their separation from New-World monkeys [1,2,21,22]. This inactivation in ancestral apes and humans was caused by a nonsense mutation of a single base deletion that introduced a premature stop codon [21,22], resulting in the inactivation of α1,3GT by truncation [23,24]. It is postulated that once primates lost the α-gal epitope, they lost their immune tolerance to it and started producing the natural anti-Gal antibody [25]. This assumption is supported by the present-day example of pigs with an inactivated (i.e., knocked out) *GGTA1* gene [26]. The synthesis of α-gal epitopes in all non-primate mammals including wild-type pigs suggests that pigs and their evolutionary lineages synthesized α-gal epitopes since early evolutionary times and, thus, did not produce anti-Gal. However, once the two *GGTA1* alleles were knocked out 23 years ago [27,28], the knockout pigs produced the natural anti-Gal antibody, similar to the production of this antibody in humans [26,29,30].

The absence of α-gal epitopes in Old-World primates vs. its synthesis in lemurs and New-World monkeys suggests that there was an evolutionary elimination of parental Old-World primates synthesizing α-gal epitopes and a survival of few primates lacking this epitope and producing the natural anti-Gal antibody [25]. It is possible that this event was associated with the processes of carbohydrate chain synthesis on enveloped viruses. This synthesis is mediated by the host–cell glycosylation machinery which is taken over by infecting viruses. Thus, enveloped viruses replicating in cells containing active α1,3GT present α-gal epitopes on their carbohydrate chains [31,32,33,34,35,36].

It is suggested that the epidemics of lethal viruses in the Eurasia–Africa landmass caused the extinction of parental Old-World primates synthesizing α-gal epitopes [25]. Among these parental primates there were few mutated offspring that carried two inactivated *GGTA1* genes, due to an accidental mutation that happened in earlier generations. Mutated primates with two inactivated *GGTA1* alleles did not synthesize α-gal epitopes and produced the natural anti-Gal antibody. The lethal viruses replicating in parental hosts containing active α1,3GT presented α-gal epitopes on their envelope. Although these viruses killed the parental hosts, they could not kill the mutated offspring because they were destroyed by anti-Gal produced by the offspring lacking α-gal epitopes. Thus, the original Old-World primates synthesizing α-gal epitopes were replaced by small populations of anti-Gal-producing mutated offspring, which survived and expanded [25]. Ancestral lemurs and New-World monkeys were not subjected to these evolutionary pressures because they were isolated from the Eurasia–Africa landmass by oceanic barriers. This assumption is supported by the reports on the anti-Gal-mediated killing of viruses presenting α-gal epitopes that were incubated in human serum at 37 °C [33,34,35,36,37]. This virolysis is mediated by activation of the complement system following α-gal/anti-Gal interactions, which result in the formation of “holes” in the viral envelope by assembled C5-9 proteins of the complement system. It is further assumed that this anti-Gal-mediated killing of viruses presenting α-gal epitopes functions at present as a first line of defense in humans against infections by zoonotic enveloped viruses which originate in non-primate mammals such as pigs, cows, sheep, goats, cats and dogs [38,39].

## 2. Characteristics of α-Gal/Anti-Gal Interaction That Enable Clinical Exploitation

There are four established immunological outcomes of α-gal/anti-Gal interactions: 1. activation of the complement system for complement-mediated cytolysis (CDC) of cells presenting α-gal epitopes; 2. localized recruitment of macrophages and dendritic cells by the chemotactic complement cleavage peptides C5a and C3a generated as a byproduct of the complement activation; 3. antibody-dependent cell cytolysis (ADCC) mediated by macrophages and natural killer (NK) cells attaching to the Fc portion (“tail”) of anti-Gal bound to cells presenting α-gal epitopes; and 4. phagocytosis of anti-Gal-coated (opsonized) cells and particulate materials presenting α-gal epitopes by macrophages and dendritic cells functioning as antigen-presenting-cells (APCs), following interaction between the Fc portion of anti-Gal and Fc receptors on these phagocytic cells.

These characteristics of anti-Gal can be demonstrated in vitro with human serum anti-Gal and in vivo with mice referred to as GT-KO mice, in which the *GGTA1* gene was knocked out. GT-KO mice do not synthesize the α-gal epitope [40]. These mice do not produce anti-Gal because they are kept in sterile conditions, which do not enable the development of the required gastrointestinal flora for production of this antibody. However, GT-KO mice produce anti-Gal following immunization with rabbit or pig cells or cell membranes presenting α-gal epitopes [41,42,43], or with glycoproteins carrying α-gal epitopes on their carbohydrate chains [44].

### 2.1. Anti-Gal-Mediated Cytolysis of Cells Presenting α-Gal Epitopes

The full potential of complement activation and cell lysis following the binding of anti-Gal to α-gal epitopes on cells was observed in the field of xenotransplantation, in which pig organs are transplanted into monkeys producing anti-Gal. The transplantation of a porcine heart or kidney into Old-World monkeys results in hyperacute rejection within 30 min to a few hours. Anti-Gal binds to the multiple α-gal epitopes on porcine endothelial cells lining the blood vessels of the xenograft (~10^7^ epitopes/cell) and activates the complement system which bores holes in the endothelial cell membranes, resulting in the collapse of the vascular bed [45,46,47]. The removal of anti-Gal from the blood by α-gal epitope columns, or its blocking by free α-gal epitopes, prevents this hyperacute rejection for a limited time till the active antibody reappears in the circulation [48,49]. Similar anti-Gal-induced cytolysis was observed in vitro when mouse or porcine cells expressing α-gal epitopes were incubated in human serum [50,51,52].

### 2.2. Localized Recruitment of Macrophages by Complement Cleavage Chemotactic Peptides

**Figure 2:**
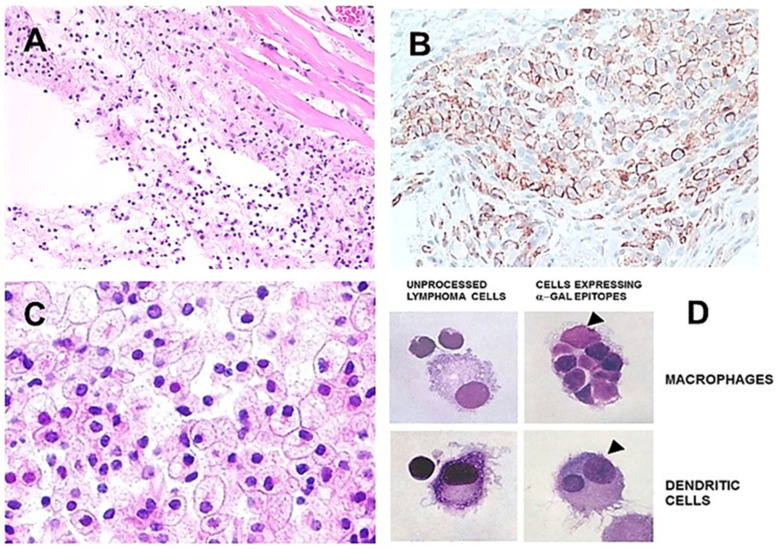
Recruitment of macrophages by α-gal/anti-Gal interaction and anti-Gal-mediated opsonization of tumor cells for uptake by macrophages and dendritic cells. (**A**) Recruitment of macrophages following intradermal injection of α-gal nanoparticles, 24 h post-injection. The empty area on the left represents the injected suspension of nanoparticles (dissolved by alcohol in staining) (H&E ×100). (**B**) Injection site after 4 days. Macrophages are immunostained specifically with HRP-anti-F4/80 antibody (×200). (**C**) Injection site after 7 days (H&E ×400). (**D**) In vitro phagocytosis by the human APCs macrophage and dendritic cell of autologous human B-lymphoma cells, engineered to present α-gal epitopes and opsonized by anti-Gal. Lymphoma cells lacking α-gal epitopes do not bind anti-Gal and are not phagocytosed. Triangles—APC nuclei. Giemsa (×1000). (**A**–**C**) Adapted from Ref. [16] with permission. (**D**) Adapted from Ref. [54] with permission.

The complement cleavage peptides C5a and C3a are byproducts of the complement activation process following α-gal/anti-Gal interaction. These peptides are among the most potent physiological chemotactic factors which induce the extensive recruitment of macrophages to the site of complement activation. This recruitment could be demonstrated by intradermal injection of α-gal nanoparticles in anti-Gal-producing GT-KO mice. The α-gal nanoparticles are small liposomes presenting multiple α-gal epitopes, which are further described below in the section on the anti-Gal-mediated regeneration of skin wounds and injured myocardium. Within 24 h post-administration of α-gal nanoparticles, a distinct migration of macrophages is observed at the injection site (Figure 2A). The number of macrophages increases after 4 days (Figure 2B) and peaks on day 7 (Figure 2C). The identity of the infiltrating cells as macrophages is confirmed by their staining with the macrophage-specific anti-F4/80 antibody (Figure 2B) [53]. After 21 days the macrophages completely disappear without changes in the normal skin structure.

### 2.3. Uptake of Cells Opsonized by Anti-Gal into Macrophages and Dendritic Cells

The Fc “tail” of anti-Gal IgG bound to α-gal epitopes on cells, or on other particulate materials, binds effectively to Fcγ receptors (FcγR) on APCs such as macrophages and dendritic cells. This binding is followed by phagocytosis of the opsonized cells by the APCs, as demonstrated with human lymphoma cells presenting α-gal epitopes (Figure 2D). Cancer cells from a lymphoma patient were engineered to present α-gal epitopes by incubation with neuraminidase, recombinant (r)α1,3GT and UDP-Gal [54,55]. α-Gal-presenting or -lacking cancer cells were co-incubated with anti-Gal, and with macrophages or dendritic cells, all obtained from the same patient. Within 2 h of incubation at 37 °C the macrophages phagocytosed as many as nine anti-Gal-opsonized lymphoma cells (Figure 2D) [54]. Lymphoma cells lacking α-gal epitopes were not phagocytosed since they did not bind anti-Gal. These anti-Gal-opsonized lymphoma cells were also internalized by dendritic cells, albeit at a lower level than the uptake observed in macrophages. A similar effective anti-Gal-mediated uptake was reported with human leukemia [55], lung and colon carcinoma cells [56], all engineered to present α-gal epitopes. As shown below, this effective anti-Gal-mediated targeting of cells and viruses for robust uptake by APCs is of great significance in the conversion of cancer cells into vaccines against tumor antigens (TAs) and in amplifying the effectiveness of viral vaccines.

## 3. α-Gal/Anti-Gal Interaction Converts Tumors into Autologous Anti-TA Vaccines

### 3.1. Conversion of Cancer Cells into Anti-TA Vaccines

In cancer patients, the relapsing disease following resection of the primary tumor is usually the result of the failure of immune-system T-cells to detect and destroy metastatic cancer cells presenting TAs. Many of these TAs are generated by accidental mutations occurring in cancer cells due to genomic instability of the cells. The TAs are the mutated proteins in cancer cells and most are unique to the individual patient [57,58,59]. If the immune system is “nimble” enough to detect the TAs within the cancer patient, metastatic cancer cells may be destroyed by cytotoxic T lymphocytes (CTL) reacting specifically against cancer cells presenting the TAs, thereby improving the prognosis of the patient [60,61]. Thus, one of the major challenges in cancer therapy is the induction of a protective CTL response against the TAs in the individual patient in whom the immune system is “oblivious “ to TAs of the patient’s cancer cells.

The detection of TAs within tumor lesions by TA-specific T-cells residing in lymph nodes requires the involvement of APCs like dendritic cells and macrophages, functioning as the “intelligence core” of the immune system. Activation of TA-specific T-cells can be achieved if the APCs infiltrate into tumors and internalize (i.e., phagocytose) cancer cells and cell membranes containing the TAs [57,62]. The APCs process the internalized self and common TAs into peptides and transport the processed TAs to regional lymph nodes [62]. In the lymph nodes, TA peptides are presented on human leukocyte antigen (HLA) molecules of APCs in a form that activates TA-specific T-cells. The activated T-cells proliferate, mature into TA-specific CTLs that leave the lymph nodes, circulate in the body, and seek and destroy cancer cells presenting the TAs [57]. Thus, achieving an effective anti-TA immune response requires two key steps: extensive infiltration of APCs into tumors and robust phagocytosis of cancer cells and cell membranes by APCs. Both these activities can be effectively induced by engineering cancer cells in treated patients to present α-gal epitopes.

**Figure 3:**
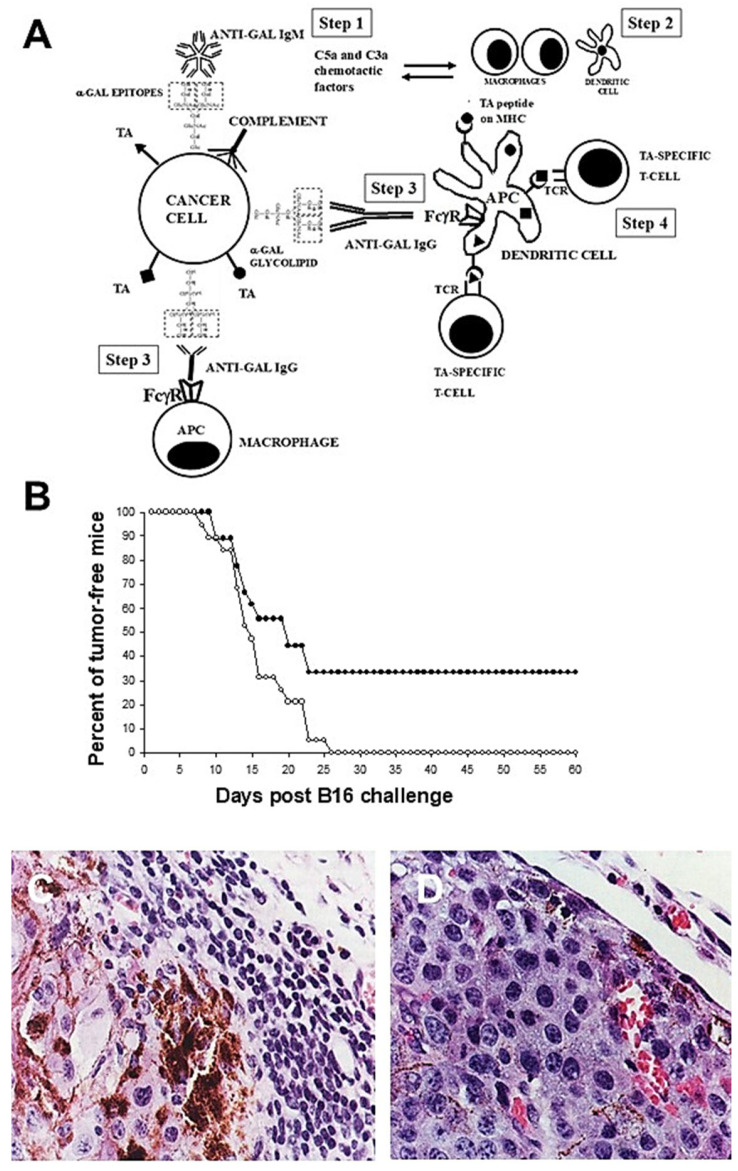
Conversion of cancer cells into vaccine against autologous TAs. (**A**) Illustration of the hypothesis on the steps occurring following in situ expression of α-gal epitopes on cancer cells (see text). Carbohydrates in rectangles represent α-gal epitopes. (**B**) Protection against development of B16 melanoma lesion in anti-Gal-producing mice immunized with irradiated B16_α-gal_ cells (●) or B16 cells (o) and challenged with live B16 cells (n = 27/group). (**C**) Histology of melanoma lesions developing in mice immunized with B16_α-gal_ cells. (**D**) Histology of melanoma lesions developing in mice immunized with B16 cells. Adapted from Ref. [16] with permission.

It has been postulated that in situ binding of anti-Gal to cancer cells engineered to present α-gal epitopes will initiate a sequence of events described as the following steps (Figure 3A) [55,63]: 1. The α-gal/anti-Gal interaction will activate the complement system, which kills opsonized cancer cells and generates complement cleavage chemotactic peptides C5a and C3a. 2. These peptides will induce extensive recruitment of APCs into the treated tumor. 3. The recruited APCs will bind to cancer cells or cell membranes coated (opsonized) by anti-Gal via Fc/FcγR interaction, phagocytose and transport them to regional lymph nodes. This binding and uptake by APCs of cancer cells presenting α-gal epitopes will be further augmented by an interaction between the complement component C3b on these cells and complement receptor 1 (CR1) on APCs. 4. In the lymph nodes, the APCs will present processed TAs of internalized cancer cells and activate the TA-specific T-cells. These cells will proliferate and differentiate into CTLs that leave the lymph nodes to seek and destroy cancer cells presenting the TAs.

Anti-TA tumor vaccines presenting α-gal epitopes were first studied in anti-Gal-producing GT-KO mice, with the mouse B16 melanoma cells which lack α-gal epitopes (most other mouse cancer cells present α-gal epitopes) as the tumor model [64]. B16 cells are highly tumorigenic, develop tumor lesions which double their size every 4–8 days, and display low immunogenicity. B16 cells were engineered to present multiple α-gal epitopes by stable transfection with the *GGTA1* gene. Anti-Gal-producing GT-KO mice were immunized with 2 × 10^6^ lethally irradiated B16 cells or irradiated B16 cells presenting α-gal epitopes (referred to as B16_α-gal_ cells). Fourteen days later, mice were challenged subcutaneously with 0.5 × 10^6^ live B16 cells and tumor development (≥5 mm) was monitored. All mice immunized with B16 cells developed tumors within ≤26 days, whereas 36% of mice immunized with B16_α-gal_ cells did not develop any tumor lesion (Figure 3B) [64]. Similar results, conferring protective immunogenicity to TAs of B16, were demonstrated in an independent study in which B16 cells were engineered to present α-gal epitopes by transduction of the *GGTA1* gene with a retrovirus [65]. Similarly, the expression of α-gal epitopes on immunizing pancreatic cancer cells was found to markedly increase their TA immunogenicity in the GT-KO mouse model [66].

The histology of melanoma lesions in mice immunized with B16_α-gal_ cells and challenged with live B16 cells demonstrated mononuclear cells surrounding the lesions (Figure 3C). These inflammatory cells comprised T-cells and macrophages that killed cancer cells or stopped cancer-cell division in the external areas of the lesions, indicated by intra-cellular vacuoles and the production of melanin, respectively [64]. These findings suggested that, in B16_α-gal_-immunized mice which developed melanoma lesions, the immune-system cells, activated against B16 TAs, failed to penetrate the lesion, possibly because of an immunosuppressive microenvironment in it. In contrast, lesions in mice immunized with B16 cells (lacking α-gal epitopes) displayed no development of a protective immune response against the challenging cancer cells and the tumors were surrounded only by a thin connective tissue layer (Figure 3D).

These findings implied that engineering cancer cells to present α-gal epitopes increases their TA immunogenicity and can result in the induction of a protective immune response, potent enough to prevent tumor growth following a challenge that killed 100% of mice immunized with the same cancer cells lacking α-gal epitopes. These observations further prompted the development of methods for in situ expression of α-gal epitopes on cancer cells in patients, thereby converting these cells into vaccines against autologous TAs.

### 3.2. Intra-Tumoral Injection of α-Gal Glycolipids

**Figure 4:**
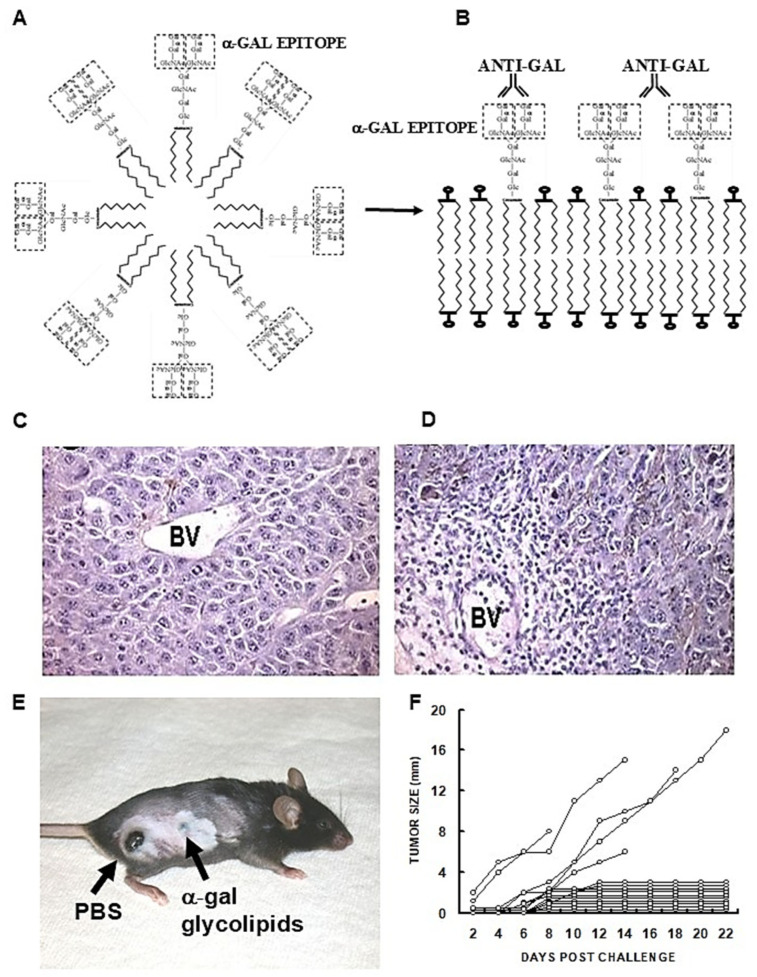
Therapeutic effects of α-gal glycolipids in mouse B16 melanoma. (**A**) Schematic illustration of an α-gal glycolipid micelle. The 3D shape is of a sphere. (**B**) Intra-tumoral injection of α-gal glycolipid micelles results in spontaneous insertion of the lipid tails of these glycolipids into the lipid bi-layer of tumor cells and expression of α-gal epitopes on the cell membranes. These epitopes readily bind anti-Gal. (**C**) A section of B16 lesion in anti-Gal-producing GT-KO mice, 4 days post-intra-tumoral injection of PBS (H&E ×100). (**D**) A section of B16 lesion, as in (**C**), 4 days post-intra-tumoral injection of α-gal glycolipids. Note the many macrophages extravasating a blood vessel (BV) (H&E ×100). (**E**) Two B16 lesions (5 mm diameter) injected with PBS or with α-gal glycolipids and viewed 10 days post-injection. (**F**) Growth of 10^4^ B16 cells in the left flanks of mice into ≥5.0 mm lesions following injection of α-gal glycolipids into the primary lesion in the right flank. Note that no tumor growth was observed in 10 of the 15 mice studied. Adapted from Ref. [16] with permission.

The first method used for in situ expression of α-gal epitopes on cancer cells was intra-tumoral injection of α-gal glycolipids [67]. These glycolipids are extracted from rabbit red cell membranes incubated in chloroform:methanol 1:2 (illustrated in Figure 1), and the removal of the phospholipids and cholesterol by preferential solubility of the α-gal glycolipids in an aqueous solution (Folch partition). When dissolved in saline, these glycolipids form micelles (Figure 4A). The injection of such α-gal glycolipid micelles into tumors results in a spontaneous insertion of the α-gal glycolipids into cancer-cell membranes, because their hydrophobic lipid “tail” is energetically much more stable when surrounded by cell membrane phospholipids than in micelles surrounded by water molecules (Figure 4B). The extensive recruitment of macrophages following the α-gal/anti-Gal interaction within tumors injected with α-gal glycolipids is clearly observed around blood vessels within 4 days (Figure 4D). No macrophage infiltration was detected in tumors injected with PBS (Figure 4C).

The efficacy and safety of the α-gal glycolipid therapy was tested in anti-Gal-producing GT-KO mice injected subcutaneously in the abdominal flank with 10^6^ B16 melanoma cells at two sites ~3.0 cm apart. The cells grew into two lesions each of ~5 mm diameter within 5 days. One of these lesions was injected with 1.0 mg α-gal glycolipids and the second with PBS. Within the following 10 days, the PBS-injected lesion increased its size to 12 mm whereas the α-gal glycolipid-injected tumor regressed to a diameter of ~1.5 mm (Figure 4E). This tumor regression was the result of combined CDC and ADCC, mediated by anti-Gal binding to α-gal epitopes on the tumor cells [67].

Intra-tumoral injection of α-gal glycolipids into B16 tumors further induced a systemic protection against the TAs of this melanoma. This was demonstrated in anti-Gal-producing GT-KO mice, which received in one flank 10^6^ B16 cells (simulating the primary tumor) and in the contra-lateral flank 10^4^ B16 cells (simulating a distant 100-fold-smaller metastasis). The primary tumors were injected with 1.0 mg α-gal glycolipids and the development of the distant metastasis was monitored for 22 days. In 66% of the mice, the 10^4^ B16 cells in the contra-lateral flank failed to progress and form visible melanoma lesions due to the induced anti-TA immune protection (Figure 4F). In contrast, in all mice with primary tumors injected with PBS instead of α-gal glycolipids, the 10^4^ B16 cells in the contra-lateral flank developed within 22 days into melanoma lesions with a size of ≥5.0 mm [67]. These findings implied that the conversion of the primary tumor into an anti-TA vaccine induced, in most of the treated mice, a protective immune response potent enough to destroy distant metastatic cells and prevent them from proliferating into detectable lesions. This protective effect of the immune system was prevented if CD8^+^ T-cells (i.e., cells maturing into CTLs following activation) were eliminated [68]. A similar conversion of B16 cells into vaccines was subsequently demonstrated using synthetic α-gal glycolipids [56]. It is of note that the progression rate of tumors in humans is much slower than that of B16 melanoma in mice. Thus, an induced anti-TA immune response in humans can react against tumor lesions for longer periods than against B16 melanoma in mice.

Based on the findings above and the observations indicating that the α-gal glycolipid treatment is safe, this α-gal therapy was studied in a Phase 1 clinical trial [69]. Eleven cancer patients with solid tumors, at advanced stages of the disease, received intra-tumoral injections with 0.1 mg, 1 mg, or 10 mg α-gal glycolipids in 1 mL. Five of the treated patients displayed long survival. In four of the patients, the time to progression of the disease was prolonged in comparison to historic controls. The treatment with α-gal glycolipids was found to be well tolerated and safe, as no toxicity or autoimmune reactions were observed following the intra-tumoral administration of α-gal glycolipids [69].

### 3.3. Virotherapy by Oncolytic Viruses Containing the GGTA1 Gene

A second method for achieving expression of α-gal epitopes on cancer cells in humans is virotherapy by oncolytic viruses (OVs) containing the *GGTA1* gene (OV-GT). OVs are viruses that, for several decades, were observed to replicate preferentially in cancer cells and thus destroy these cells. Some of these viruses are vaccinia virus [70,71], Newcastle disease virus [72,73], vesicular stomatitis virus [74,75] and adenovirus [76,77]. Clinical trials with several OVs reported a temporal decrease in tumor mass in many of the treated patients and even partial remission for periods of a few months to 2 years [78,79,80]. However, in a large proportion of the OV-treated cancer patients, this virotherapy is insufficient, since the protective anti-TA immune response was found to be suboptimal for achieving the effective destruction of metastatic cancer cells that were not infected by OVs [78,81,82,83,84]. Therefore, it was postulated that in situ expression of α-gal epitopes on OV-GT-infected cancer cells may improve the effectiveness of OV treatment. In addition to the anti-Gal-mediated killing of these tumor cells, the binding of anti-Gal to α-gal epitopes is likely to induce extensive recruitment of APCs to OV-GT-infected tumor cells, and robust uptake of such opsonized cancer cells and cell membranes by the APCs, ultimately resulting in effective activation of TA-specific CTLs, as illustrated in Figure 3A.

The feasibility of α-gal epitope expression on cells infected with OV-GT was first evaluated with adenovirus engineered to contain the *GGTA1* gene (Ad-GT), which introduces multiple copies of *GGTA1* into each cell. Infection of human HeLa cervical cancer cells and mouse B16 melanoma cells with Ad-GT (referred to as B16_Ad-GT_ cells) resulted in the expression of α-gal epitopes on the cell membrane within 10 h. This expression peaked to ~2 × 10^6^ α-gal epitopes per cell within 24 h [85,86]. Immunization of anti-Gal-producing GT-KO mice with irradiated B16_Ad-GT_ cells conferred resistance to the growth of B16 cells into visible tumors in 37% of mice challenged with 0.5 × 10^6^ live B16-cells, whereas only 2% of mice immunized with irradiated B16 cells containing adenovirus but lacking the *GGTA1* gene survived this challenge [86]. These studies suggested that cancer cells presenting α-gal epitopes following infection with OV-GT may serve as effective anti-TA vaccines by the mechanism described in Figure 3A [87].

The therapeutic activity of OV-GT administered intravenously was demonstrated in monkeys and humans in the seminal study of Zhong et al. [88]. The OV-GT used in that study was Newcastle disease virus (NDV) containing the *GGTA1* gene (NDV-GT). Cynomolgus monkeys (Old-World monkeys producing the natural anti-Gal antibody) were induced to develop several hepatocellular carcinoma (HCC) lesions in the liver. These lesions progressed and caused the death of all five control monkeys within 6 months. In contrast, in all five monkeys intravenously receiving NDV-GT when the HCC lesions reached the size of 1.0 cm, the lesions disappeared within 3 months. In monkeys injected intravenously with NDV lacking *GGTA1*, the lesions continued to grow in volume, albeit at a rate lower than that in the control monkeys, and two of the five monkeys died in the 6th month post-treatment. This study indicated that the destruction of HCC lesions following OV-GT virotherapy, by the combination of anti-Gal-mediated CDC, ADCC and the induction of an anti-TA protective immune response, is much more effective than the OV virotherapy without the *GGTA1* gene [87,88].

The cynomolgus monkey study was followed by Zhong et al. [88] with a Phase 1 clinical trial of NDV-GT which included 23 patients with refractory metastatic carcinomas in the liver, ovary, breast, esophagus, lung, cervix, and rectum, or with melanoma, at advanced stages of the disease. Among the 20 evaluable patients, one had complete remission, six displayed partial remission, and 11 maintained stable disease that ranged between 4 and >36 months. In only two patients, the disease progressed following the treatment. Thus, the beneficial effects of this virotherapy were observed in 90% of the patients and no severe adverse events were reported. In biopsies of lesions performed 3 months post-treatment, expression of α-gal epitopes on tumor cells, and the presence of the virus within the cells, could be demonstrated by immunostaining. Moreover, the observation of seven patients displaying partial remission or stable disease for >25 months at the time of the study report [88] strongly suggests an ongoing protective anti-TA immune response. Such an immune response prevents the progression of tumor lesions in their periphery. However, there is a limited ability of CTLs and macrophages to penetrate cores of such lesions, which may contain an immunosuppressive microenvironment, analogous to the lesion in Figure 3C. The efficacy of the NDV-GT virotherapy in cancer patients may also be affected by previous chemotherapy and radiation treatments, which impair the immune system activity in periods overlapping the NDV-GT virotherapy treatment. An analysis of anti-Gal titer and non-specific T-cell activation may provide insight into the state of the immune system prior to the virotherapy.

In view of the considerations above, which may affect the effectiveness of OV-GT virotherapy, it is suggested that this therapy may be most effective if delivered as a neo-adjuvant therapy within a few days of the detection of the primary tumor and prior to its resection. At that time, the immune-system efficacy is not impaired by other treatments, and the tumor burden and metastases number are the lowest. It is postulated that, within the period of 2–4 weeks prior to a planned resection of the primary tumor and of other detectable lesions, the APCs will be recruited into the OV-GT-infected lesion(s). These APCs will phagocytose anti-Gal-opsonized cancer cells and cell membranes and transport the internalized TAs to regional lymph nodes for activation of TA-specific T-cells, as illustrated in Figure 3A. Thus, the vaccinating effects of the cancer cells presenting α-gal epitopes will continue within the lymph nodes, even after the resection of detectable tumors.

### 3.4. In Vitro Synthesis of α-Gal Epitopes on Vaccinating Cancer Cells

An alternative approach for the in situ expression of α-gal epitopes on cancer cells has been the in vitro synthesis of this carbohydrate antigen by the use of rα1,3GT and UDP-Gal on hematological cancer cells obtained from the patient (leukemia and lymphoma cells), or on cancer cells and cell membrane homogenates prepared from solid tumors resected from the patient [55,89]. The number of synthesized α-gal epitopes can be greatly increased by the addition of neuraminidase, which removes sialic acids (SA) from many carbohydrate chains and exposes the penultimate N-acetyllactosamines (Galβ1-4GlcNAc) serving as the acceptor for rα1,3GT. After such engineering, the cancer cells or cell homogenates are irradiated and injected into the patient as anti-TA vaccines. A clinical trial performed with HCC patients included immunization with α-gal-presenting autologous cell membranes prepared from resected tumors [90]. This treatment resulted in the prolonged survival of ~70% of the treated patients, in comparison to non-immunized patients. In a second study, lymphoma patients were immunized with autologous cancer cells enzymatically engineered to present α-gal epitopes. Among 14 immunized patients, complete remission was reported in four patients, partial remission in three patients, and stable disease in five patients [91].

The few clinical trials with autologous cancer cells engineered in situ in cancer patients or in vitro to present α-gal epitopes suggest that anti-Gal-mediated exposure of the immune system to autologous TAs may be, in some patients, potent enough to induce a protective anti-tumor immune response [63]. These immunotherapies seem to be safe in treated patients. Nevertheless, much more research is needed for optimizing these α-gal therapies to maximize their effectiveness in various cancer patients.

## 4. α-Gal/Anti-Gal Interaction Amplifies Effectiveness of Inactivated Whole-Virus Vaccines

A second clinical use of the α-gal/anti-Gal interaction in humans is for increasing the effectiveness of currently used vaccines for protection against infections by enveloped viruses. Influenza (flu) vaccines are only partially effective in protecting against flu virus infections in elderly individuals. The currently used COVID-19 mRNA vaccines must be renewed with the appearance of variants with mutations in the S-protein of the virus. Such mutations enable escape from the anti-S antibodies in immunized individuals. It was postulated that inactivated whole-virus vaccines presenting α-gal epitopes (referred to as α-gal vaccines) will be much more effective in eliciting a protective immune response against various viruses than viral vaccines lacking these epitopes [92,93,94,95,96].

**Figure 5:**
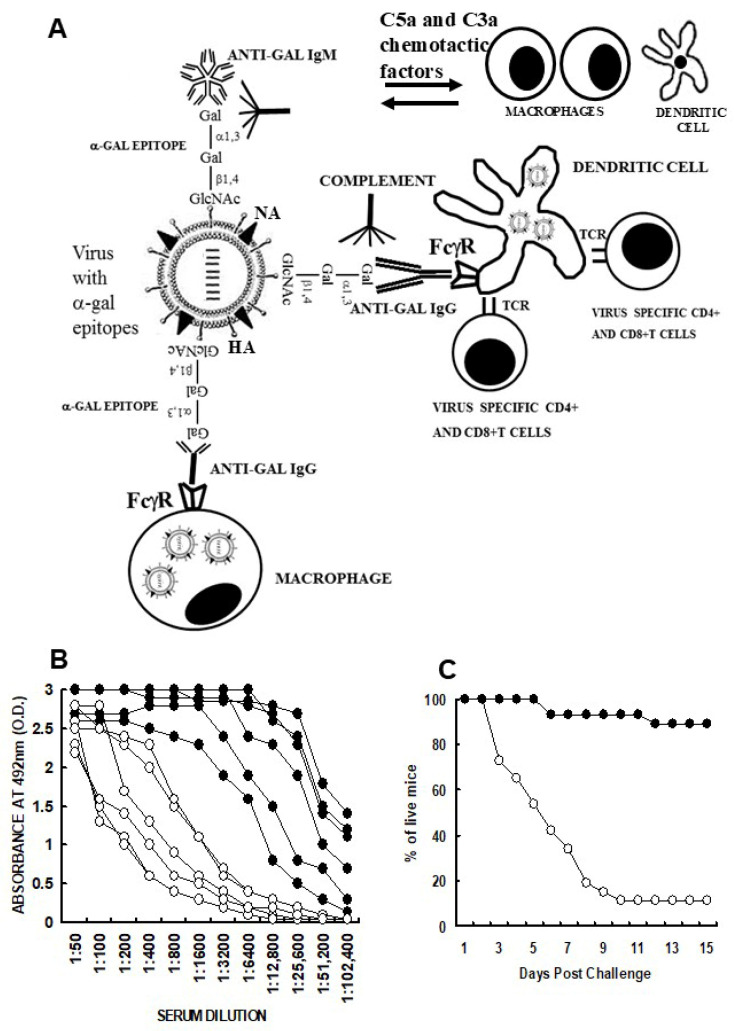
Amplification of inactivated flu whole-virus vaccine immunogenicity as a model for general immune response following immunization with α-gal vaccines. (**A**) Illustration of the hypothesized steps occurring following vaccination with flu virus engineered to present α-gal epitopes (see text). (**B**) ELISA measuring anti-PR8 IgG antibody production in anti-Gal-producing GT-KO mice receiving 2 immunizations of 1 μg inactivated PR8_α-gal_-virus (●), or of PR8-virus (o) in Ribi adjuvant. PR8 served as solid-phase antigen. (**C**) Survival of anti-Gal-producing GT-KO mice immunized with inactivated PR8_α-gal_-virus(●), or PR8-virus (o) and infected with a lethal dose of live PR8 virus. n = 25 mice per group. HA—hemagglutinin, NA—neuraminidase. (**A**) Adapted from Ref. [16] with permission. (**B**,**C**) Adapted from Ref. [94] with permission.

Vaccination with inactivated viruses presenting α-gal epitopes releases anti-Gal from ruptured capillaries at the vaccination site and is followed by binding of anti-Gal to the vaccinating virions. This α-gal/anti-Gal interaction activates the complement system and generates the complement cleavage peptides C5a and C3a that recruit many APCs to the vaccination site (Figure 5A). Anti-Gal-opsonized virions are targeted for extensive uptake by the recruited APCs, which transport such vaccines to regional lymph nodes, process and present the viral antigens for the activation of multiple helper CD4^+^ T-cells and cytotoxic CD8^+^ T-cell CTLs. Whereas the helper T-cells help B-cells to produce antibodies against the virus, the CTLs kill cells infected with the virus, thereby preventing an expansion of the infecting virus.

### 4.1. Influenza Virus Vaccine Presenting α-Gal Epitopes

The extent of the increased immunogenicity of inactivated α-gal vaccines could be assessed with the influenza virus A/Puerto Rico/8/1934 (referred to as PR8-virus). The virus, propagated in allantoic fluid of embryonated chicken eggs [92], was inactivated by 45 min of incubation at 65 °C and engineered to present α-gal epitopes (PR8_α-gal_-virus) by incubation with rα1,3GT and UDP-Gal [93]. This reaction resulted in the synthesis of ~3000 α-gal epitopes on each virion. GT-KO mice producing anti-Gal received two subcutaneous vaccinations with 1 μg PR8_α-gal_-virus or of PR8-virus in Ribi adjuvant, in 2-week interval [94]. The production of anti-PR8 antibodies, 2 weeks after the second vaccination, demonstrated a ~100-fold increase in the anti-PR8 IgG antibody titer in PR8_α-gal_-immunized mice than in PR8-immunized mice (Figure 5B) and a ~60-fold-higher anti-PR8 IgA antibody [94]. Anti-PR8 IgG antibody titers measured in mice lacking anti-Gal (i.e., wild-type mice) and immunized with PR8_α-gal_ or PR8-virus were as low as those in GT-KO mice immunized with PR8-virus (Figure 5B) [94]. This finding implies that, in the absence of anti-Gal, there were no differences in the immunogenicity of the two vaccines. A much-higher activation of flu-specific CD4^+^ and CD8^+^ T-cells was also observed in PR8_α-gal_ vs. PR8-immunized mice analyzed for IFN-γ production in ELISPOT and in flow cytometry of T-cells producing this cytokine [94]. A similar amplification of immunogenicity by α-gal epitopes was observed with the attenuated flu virus vaccine containing the *GGTA1* gene [95] and with the vaccine of recombinant gp120 of HIV engineered to present α-gal epitopes vs. the same vaccine lacking α-gal epitopes [96,97].

The difference between the abilities of PR8_α-gal_ and PR8 vaccines to elicit a protective immune response against viral infections was determined by the prevention of death in immunized mice following intranasal challenge by a lethal dose (2000 PFU) of infectious PR8-virus (Figure 5C). The survival of 88% of PR8_α-gal_-immunized mice was observed after 30 days vs. only 12% survival in PR8-immunized mice. Thus, the increased production of anti-PR8 antibodies and elevated activation of PR8-specific CTLs was further manifested in a much higher survival of mice receiving the α-gal vaccine and challenged with a lethal dose of the virus [94]. Correspondingly, the much higher anti-gp120 antibody activity in gp120_α-gal_-immunized mice effectively killed HIV in vitro, whereas anti-gp120 antibodies in gp120-immunized mice lacked this ability [96]. The observations on amplified immunogenicity with flu and gp120 α-gal vaccines suggest that this method for increased vaccine effectiveness is likely to be applicable to other inactivated enveloped virus vaccines as well.

### 4.2. COVID-19 Vaccine Presenting α-Gal Epitopes

A needed α-gal vaccine of particular significance is that of an inactivated whole SARS-CoV-2 virus vaccine presenting α-gal epitopes (SARS-CoV-2_α-gal_). Theoretically, such a vaccine may end the need for production of new mRNA vaccines following the appearance of COVID-19 variants [98]. The most common COVID-19 vaccine that is currently used is an mRNA vaccine which mediates production of the SARS-CoV-2 S-protein as a vaccinating antigen in humans. The effectiveness of this vaccine is limited because of the emergence of variant virions with escape mutations in the S-protein gene. These mutations enable the virus to evade the protective anti-S protein antibodies in individuals vaccinated with the non-mutated S-protein mRNA. Thus, such variants are a constant moving target that require the production of mRNA of S-protein vaccines containing the new mutations [99].

SARS-CoV-2 virus has several proteins that may serve as target antigens in addition to the envelope S-protein. These are the E (envelope) and M (membrane) proteins on the viral envelope and the N (nucleocapsid) protein within the virus. It is postulated that a potent inactivated SARS-CoV-2 virus vaccine can induce an effective antibody response against the E and M proteins in addition to the anti-S protein antibodies and activation of CTL that kill cells infected by the virus and presenting peptides of the N protein. The combined activity of anti-E and anti-M antibodies and the anti-N CTL may result in the destruction of S-protein variants in the absence of effective anti-S antibodies.

An available inactivated SARS-CoV-2 whole-virus vaccine was found to have suboptimal effectiveness in eliciting a protective immune response [100]. This suboptimal effectiveness is likely to be associated with the 22 N-linked carbohydrate chains on the S-protein [101] that form a dense “glycan shield”, which protects the virus from antibodies and decreases its immunogenicity [98]. In view of the ~100-fold increase in immunogenicity of the flu virus vaccine presenting α-gal epitopes, described above, it is postulated that engineering SARS-CoV-2_α-gal_ as an inactivated virus vaccine will increase its immunogenicity so that the vaccinated individuals will generate potent antibody and CTL responses against S, E, M and N viral proteins that may destroy both non-mutated infecting SARS-CoV-2 and variants mutated in the S-protein [98,99].

### 4.3. Methods for Engineering Virus Vaccines Presenting α-Gal Epitopes

Some of the methods for the preparation of vaccinating inactivated enveloped viruses presenting α-gal epitopes are: 1. Incubation of the inactivated virus with rα1,3GT, UDP-Gal and neuraminidase (for the removal of sialic acid, not needed with flu virus) [93]. 2. Propagating the virus in cells engineered to contain several copies of the *GGTA1* gene, to produce a high concentration of α1,3GT in host cells which will synthesize α-gal epitopes on viral envelope glycoproteins. 3. Insertion of the *GGTA1* gene into the genome of the virus prepared as a vaccine [85,86,87,88,95]. *GGTA1* within the propagated virus will be translated into many copies of α1,3GT, which will synthesize multiple α-gal epitopes on envelope glycoproteins of the newly produced virions within the host cells. A high concentration of α1,3GT in the host cells is required because this glycosyltransferase competes with sialyltransferases for capping N-linked carbohydrate chains within the trans-Golgi compartment of host cells [102].

## 5. α-Gal Nanoparticles Induce Scar-Free Regeneration of Injured Wounds, Heart and Spinal Cord

Heart injuries in urodele amphibians (salamander, newt and axolotl), neonatal mice and neonatal pigs undergo complex regenerative processes which restore the normal structure and function of the injured myocardium [103,104,105,106,107]. These regenerative processes are initiated by an extensive migration of macrophages to the injury site [105,108,109] and the innate activation of components of the complement system [110]. In contrast, injury of the myocardium in mice older than one week and in adult mammals (e.g., ischemia following myocardial infarction [MI]) is repaired by fibrosis and scar formation in the ventricular wall of the heart [111]. In adult mice and humans, this repair mechanism prevents a rupture of the ventricular wall, but the impaired contractility of the heart can lead to heart failure and premature death [111,112].

The macrophage-mediated regeneration in urodeles and neonatal mice vs. the macrophage repair by fibrosis and scar formation in adult mice led to the assumption that the macrophage regenerating mechanism in urodeles and neonatal mammals is conserved in a suppressed form in adult mammals, and the manipulation of macrophages may lead to its re-activation [105,108,109,113]. Thus, it was of interest to determine whether complement involvement in the recruitment of macrophages to the injury site by α-gal/anti-Gal interaction, using α-gal nanoparticles, and the subsequent binding of anti-Gal-coated α-gal nanoparticles to the FcγR on the macrophages can modulate these cells in adult mice. Such theoretical modulation was tested for inducing polarization of the recruited macrophages into pro-regenerative macrophages that orchestrate a regeneration of the injured tissue, instead of the default polarization into pro-reparative macrophages that induce fibrosis and scar formation.

### 5.1. In Vivo Effects of α-Gal Nanoparticles

**Figure 6:**
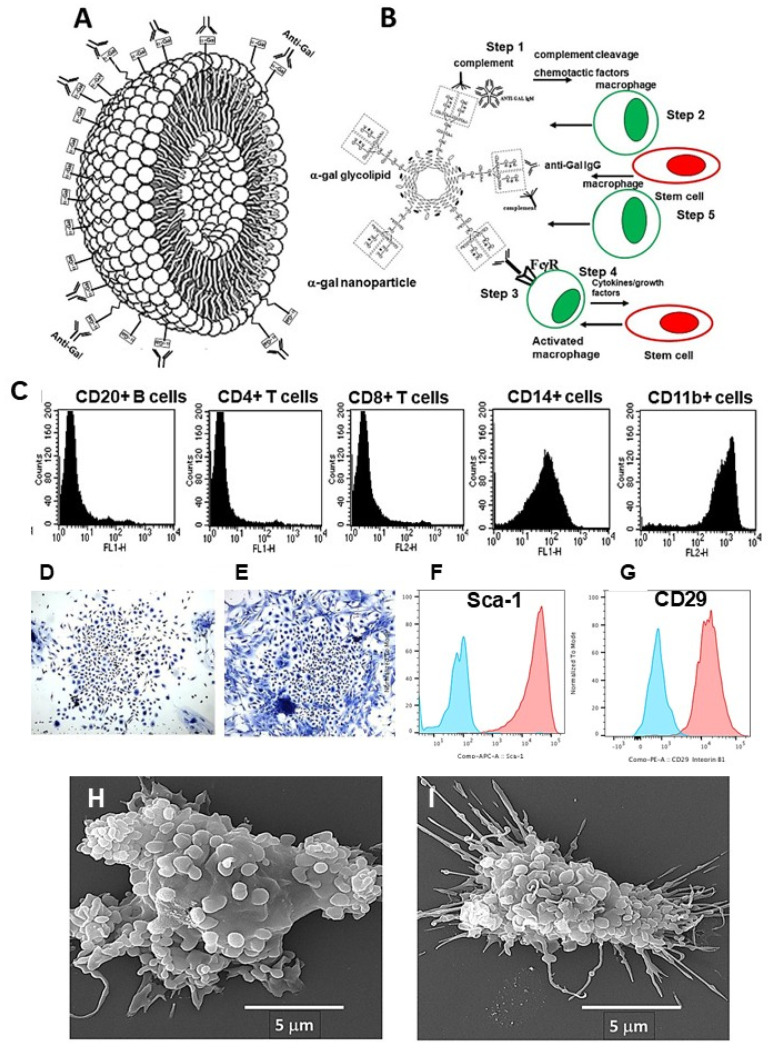
Characteristics of α-gal nanoparticles. (**A**) α-Gal nanoparticles (~0.1–0.3 μm) and α-gal liposomes (≥0.5 μm) are small spheres made of a phospholipid bi-layer and α-gal glycolipids anchor in it by the lipid tail. Anti-Gal IgG and IgM molecules specifically bind to the α-gal epitopes (rectangles) on liposomes/nanoparticles. (**B**) Sequence of steps occurring following administration of α-gal nanoparticles or α-gal liposome into injuries (see text). (**C**) Flow cytometry of cells recruited for 7 days into PVA sponge discs containing 10.0 mg α-gal nanoparticles indicate that these cells are macrophages (CD11b^+^ and CD14^+^). No significant numbers of T- or B-cells are detected. (**D**,**E**) Cell colonies which appear following 5-day culture of the cells migrating into PVA sponge discs containing α-gal nanoparticles. (**F**,**G**) Cells harvested from colonies as in (**D**,**E**), display by flow cytometry the stem cell markers Sca-1 and CD29. Blue- isotype control; orange- marker staining. (**H**,**I**) Scanning electron microscopy of two macrophages with α-gal nanoparticles binding to them via Fc/FcγR interaction following 2 h incubation of the macrophages with anti-Gal-coated α-gal nanoparticles. Adapted from Ref. [118] with permission.

α-Gal nanoparticles are small biodegradable spheres (~0.1–0.3 μm) with a wall made of phospholipids, cholesterol and multiple α-gal glycolipids, all extracted from rabbit red cell membranes (Figure 1A and Figure 6A) [114,115,116]. The extracted mixture was sonicated in saline to generate α-gal liposomes with a size of ≥0.5 μm. Additional sonication decreases the size of these liposomes to 0.1–0.3 μm, which are referred to as “α-gal nanoparticles”. It was postulated that α-gal nanoparticles applied to wounds or injected into injured tissues will bind anti-Gal released from ruptured capillaries (Step 1 in Figure 6B) [114,115,116]. This interaction will induce localized activation of the complement system within the injury site, resulting in the production of the chemotactic complement cleavage peptides C5a and C3a. These chemotactic peptides will induce rapid and extensive migration of macrophages into the injury site (Step 2) and binding of the anti-Gal-coated α-gal nanoparticles to these macrophages via Fc/FcγR interaction (Step 3). The bound α-gal nanoparticles were expected to be phagocytosed by the macrophages as a result of this interaction, as in Figure 2D. Studies were performed in anti-Gal-producing GT-KO mice for characterizing these macrophages and determining whether they can display a pro-regenerative activity by secreting pro-regenerative cytokines and by recruiting stem cells (Step 4).

The characterization of macrophages recruited by α-gal nanoparticles was achieved by implanting, subcutaneously in the mice, biologically inert sponge discs made of polyvinyl alcohol (PVA) and containing ~10 mg α-gal nanoparticles. The sponge discs were retrieved after 7 days and were found to contain ~0.5 × 10^6^ macrophages (CD14^+^ and CD11b^+^) and very few other white cells (Figure 6C). The macrophages displayed the M2 markers IL10 and arginase-1 and lacked the M1 marker IL12 [117]. Incubation of these macrophages for 5 days resulted in the appearance of several cell colonies similar to colonies formed by cultured stem cells (Figure 6D,E) [117]. The cells in these colonies displayed the mesenchymal stem-cell markers Sca-1 and CD-29 (Figure 6F,G, respectively) [118]. When anti-Gal-coated α-gal nanoparticles were co-incubated with macrophages for 2 h, they readily adhered to the macrophages via the Fc/FcγR interaction (Step 3) as shown by scanning electron microscopy in Figure 6H,I. This interaction was further found to induce vascular endothelial growth factor (VEGF) secretion from the macrophages [115]. All these findings supported the assumption in Figure 6B that the recruitment of stem cells and regenerative processes may be mediated by the putative pro-regenerative macrophages recruited into injuries by α-gal nanoparticles. Stem cells reaching various tissues receive “cues” from healthy cells and the microenvironment, directing them to differentiate into cells that regenerate the injured tissue. Thus, it was suggested that stem cells recruited by the pro-regenerative macrophages migrating into α-gal nanoparticle-treated injured tissues might contribute to regenerative processes in adult mice [117]. These putative healing and regenerative effects were first studied in skin injuries of anti-Gal-producing GT-KO mice.

### 5.2. Healing of Skin Burns and Wounds

**Figure 7:**
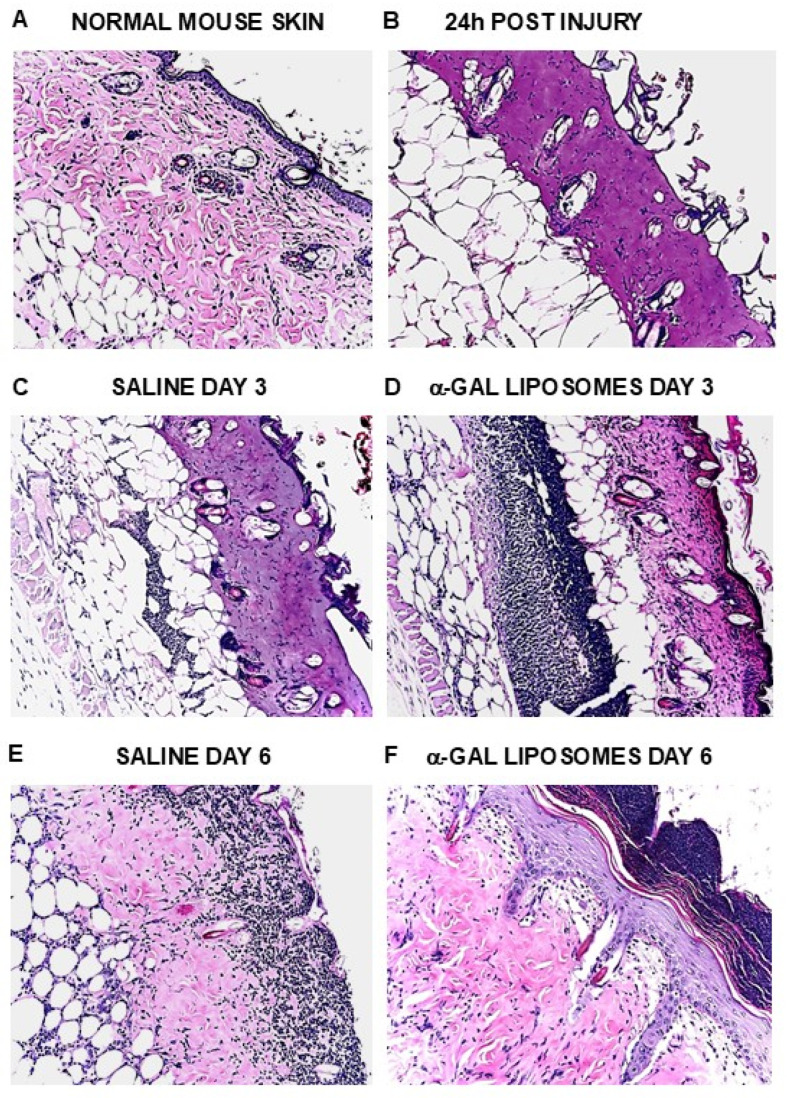
Accelerated healing of burns treated with α-gal liposomes. The burn is analogous to a second-degree burn in humans in which the burn damages the epidermis and the outer half of the dermis. On day 3, the number of macrophages recruited into the burn site is much higher in α-gal liposome-treated burns than in saline-treated burns. On day 6, the burn is completely healed in the α-gal liposome-treated burn with the eschar above the healed skin, whereas the saline-treated burn displays no epidermis regrowth and macrophages accumulating in the exposed dermis (H&E ×100). Reprinted from Ref. [16] with permission.

The in vivo effects of α-gal liposomes/nanoparticles in injury sites were first tested in thermal skin injuries. Second-degree 2 × 3 mm burns were applied by a brief touch of the skin with the heated metal end of a spatula in anesthetized anti-Gal-producing GT-KO mice. The application of wound dressing containing 10 mg α-gal liposomes to such skin burns resulted in accelerated recruitment of macrophages to the injured skin, clearly observed by day 3 (Figure 7) and further healing of the burns (i.e., covering the burn with regenerating epidermis) within 6 days post-injury [114]. Control wounds treated with saline displayed lower macrophage recruitment by day 3 (Figure 7) and full regrowth of the epidermis within 12–15 days [53,114]. Accelerated healing was also observed in skin thermal injuries caused by irradiation [119].

**Figure 8:**
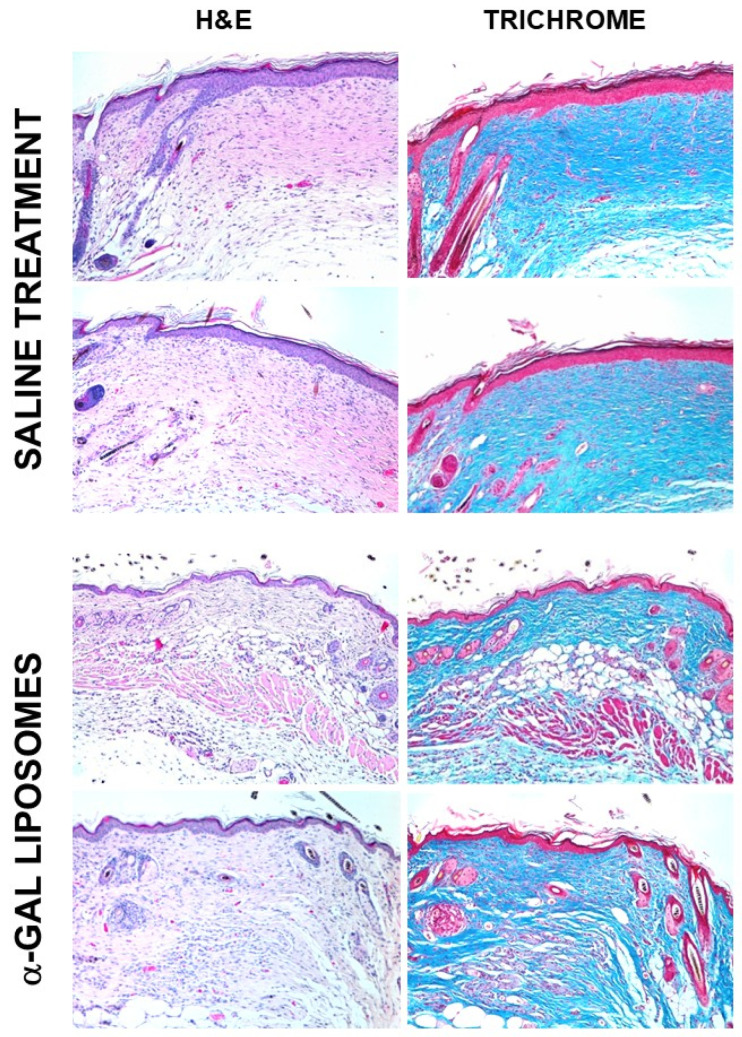
Scar-free regeneration of α-gal liposome-treated wounds. Two representative wounds treated with α-gal liposomes and those treated with saline, 28 days post-treatment. The saline-treated wounds display scar formation of hypertrophic epidermis, dense connective tissue in the dermis (deep blue staining of collagen by Masson’s trichrome) and complete absence of hair shafts, adipocytes and smooth muscles. In contrast, α-gal liposome-treated wounds display normal skin structure, with thin epidermis, loose connective tissue in the dermis and appearance of hair shafts, adipocytes and smooth muscle cells. Reprinted from Ref. [16] with permission.

Similar treatment of oval full-thickness wounds (~6 × 9 mm) with wound dressing containing 10 mg α-gal liposomes also resulted in healing by day 6, whereas saline-treated wounds healed only after 12–15 days [53,115,120]. Inspection of the wounds 28 days post-treatment demonstrated, in control wounds, the results of the default healing mechanism characterized by a hyperplastic epidermis, dense fibrosis and scar formation (Figure 8). In contrast, α-gal liposome-treated wounds displayed restoration of the normal skin structure in the thin epidermis, loose connective tissue, and the appearance of skin appendages such as hair shafts, smooth muscle cells and adipocytes (Figure 8) [53,115]. Repeated studies with α-gal nanoparticles instead of α-gal liposomes demonstrated a higher efficacy of the nanoparticles in comparison to the liposomes, possibly because of the better distribution of the smaller nanoparticles in the treated wounds [115].

These findings suggested that the accelerated healing induced by macrophages recruited by α-gal liposomes occurs before the initiation of the default repair mechanism of fibrosis and scar formation; thus, the restoration of normal skin structure prevents the healing by default fibrosis and scar formation. Effective wound healing by α-gal nanoparticles was also observed in chronic wounds of diabetic GT-KO mice. The application of α-gal nanoparticles to such wounds resulted in their healing by 18 days, whereas saline-treated wounds in the diabetic mice did not heal at all [53,121]. Accelerated wound healing by α-gal nanoparticles was also observed in 2 × 2 cm wounds in *GGTA1* knockout pigs which produce the natural anti-Gal antibody [122].

The mechanism of accelerated scar-free regeneration of skin injuries treated with α-gal liposomes/nanoparticles requires further elucidation. Preliminary studies suggest that macrophages recruited by, and interacting with, α-gal nanoparticles are polarized to secrete pro-regenerative cytokines such as VEGF, various growth factors [115] and stem cell-recruiting cytokines [118]. Overall, the findings of accelerated scar-free healing suggest that the innate regenerative mechanism observed in urodeles and in neonatal mice is reactivated by this treatment in adult mice. This assumption is further supported by studies on the regeneration of injured hearts in adult mice, described below.

### 5.3. Post-MI Myocardial Regeneration

Studies on post-MI healing of adult mouse hearts indicated that fibrosis and scar formation are preceded by two waves of macrophage infiltration. The first wave is of macrophages that debride the injured ischemic myocardium. The second is of macrophages that orchestrate the replacement of the debrided myocardium with dense connective tissue (i.e., fibrosis), forming the scar tissue which prevents a rupture of the ventricular wall [111,112]. In order to determine whether α-gal nanoparticles can induce post-MI regeneration of the injured myocardium, instead of scar formation, the chests of anti-Gal-producing GT-KO mice were opened, and the mid-left anterior descending (LAD) coronary artery was occluded by ligation for simulating MI. After 30 min, the ligature was removed, allowing for reperfusion of the ischemic myocardium. Two 10 µL injections of α-gal nanoparticles in saline (10 mg/mL) were administered into the reperfused myocardium area and the chest was closed [116]. Control mice underwent similar occlusion/perfusion but were injected with saline, instead of α-gal nanoparticles.

**Figure 9:**
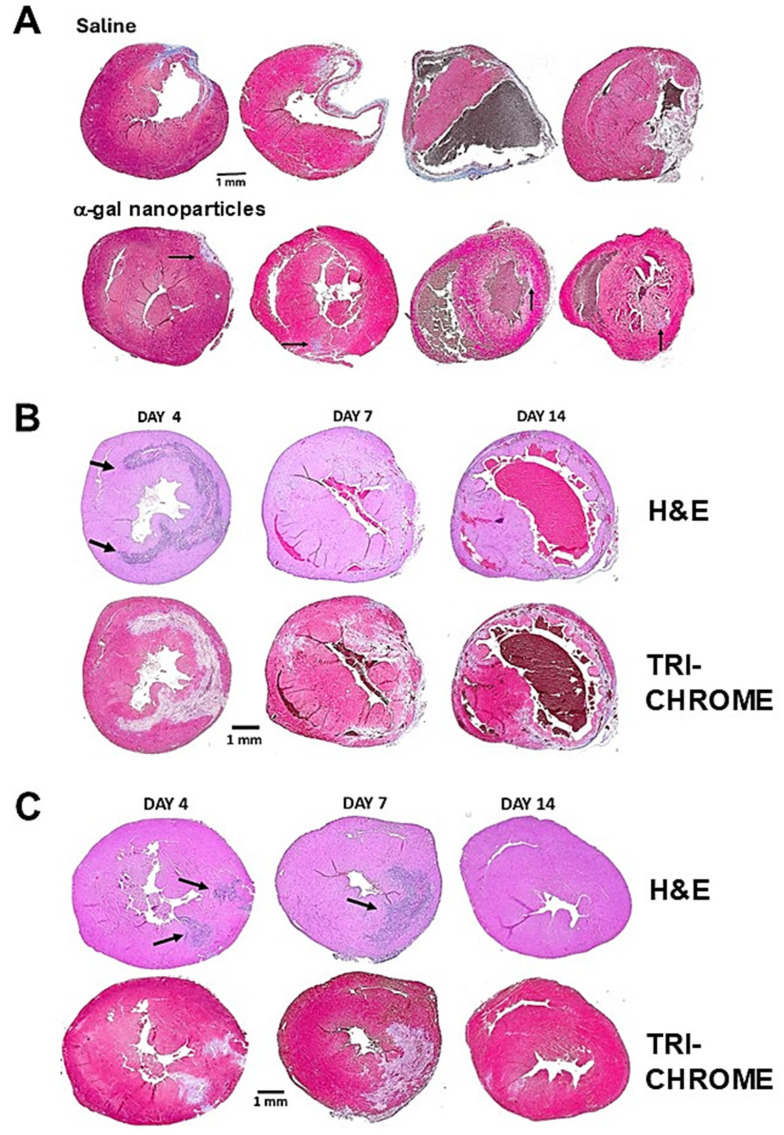
Post-MI scar formation in representative saline-treated mouse hearts and regeneration in representative hearts treated with α-gal nanoparticles. (**A**) Saline-treated (n = 10) and α-gal nanoparticle-treated hearts (n = 20) on day 28 post-MI. Fibrotic tissues stained blue/gray by Masson’s trichrome in saline-treated hearts and residual blue/gray fibrotic tissue marked with arrows in α-gal nanoparticle-treated hearts. (**B**) Post-MI saline-treated hearts on days 4, 7 and 14. H&E staining for identification of infiltrating macrophages (marked with arrows). Masson’s trichrome staining of debrided myocardium—white/light gray; red cells—brown; and healthy myocardium—red. Fibrosis initiated on day 14 and the ventricular wall is thinning. (**C**) Post-MI α-gal nanoparticle-treated hearts. Staining and arrows as in (**B**). Adapted from Ref. [116] with permission.

Histological evaluation of the hearts in the two groups on day 28 post-MI indicated that the healing of reperfused hearts injected with saline resulted in scar formation in 15–35% of the ventricular wall. In contrast, all hearts injected with α-gal nanoparticles displayed a near-complete regeneration of the functional myocardium (Figure 9A) [116]. Echocardiography analysis demonstrated a marked decrease in contractile activity (i.e., fraction shortening) in both groups on day 7 post-MI due to the ischemic injuries which continued in the saline-treated group, also on day 28. However, in the α-gal nanoparticle-treated group, the contractile activity returned to the normal level, measured prior to the LAD occlusion [116]. The histology of saline-treated hearts at various time points demonstrated extensive infiltration of macrophages by day 4 (Figure 9B). Most of these cells disappeared after 7 days and, within 14 days, the ventricular wall displayed initiation of fibrosis and scar formation. In contrast, in α-gal nanoparticle-treated hearts, infiltration of macrophages on day 4 was observed in the two injections areas. This infiltration of macrophages debriding the injured myocardium peaked on day 7. By day 14, the infiltrating macrophages disappeared and the myocardium of the left ventricular wall displayed complete regeneration (Figure 9C).

### 5.4. Inactivation of Neutrophil Activity in Injured Heart by α-Gal Nanoparticles

**Figure 10:**
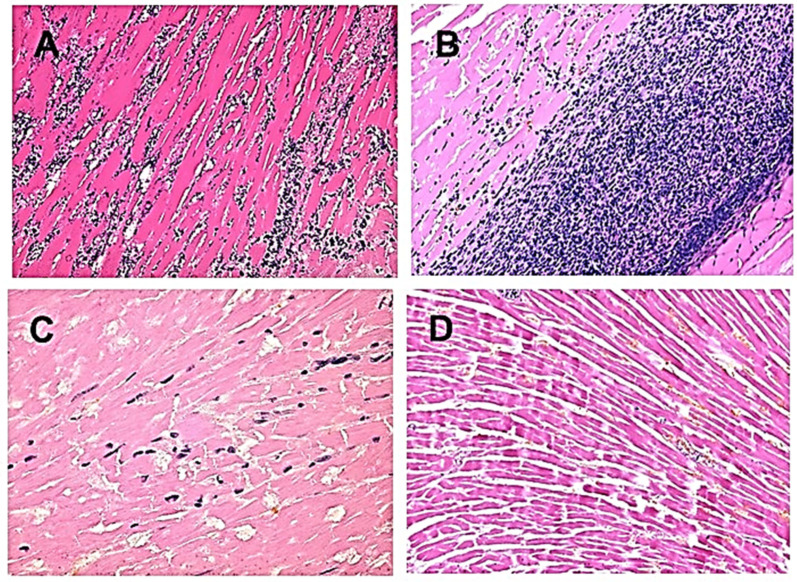
Prevention of neutrophil infiltration and delay of cardiomyocyte necrosis in dead hearts of GT-KO mice injected with α-gal nanoparticles and implanted subcutaneously in anti-Gal-producing GT-KO mice. (**A**) Heart injected with saline and implanted for 2 weeks. Note the necrotizing neutrophils and the acidophilic dead cells. (**B**) Heart injected with α-gal nanoparticles and implanted for 2 weeks. Note the extensive infiltration of macrophages at the injection site of α-gal nanoparticles. (**C**) Hearts injected with α-gal nanoparticles and implanted for 4 weeks, displaying an area containing few infiltrating macrophages far from the nanoparticle injection site. Note the conservation of the cardiomyocytes structure and the intercalated discs despite lack of blood supply and of nuclei in the cardiomyocytes. (**D**) Conservation of cardiomyocytes structure as in (**C**) despite the complete absence of macrophages. H&E ×100. Adapted from Ref. [16] with permission.

The regenerative mechanisms and the origin of cardiomyocytes that proliferate in α-gal nanoparticle-treated adult mice, during the second week post-MI, are not clear at present. The macrophages active in α-gal nanoparticle-injected hearts clearly display pro-regenerative activity, whereas those in the saline-treated heart display the default pro-reparative activity leading to scar formation. Another clear effect of the α-gal nanoparticles injected into the adult heart is on neutrophils. Hearts harvested from healthy GT-KO mice were injected in vitro with saline or with 1.0 mg α-gal nanoparticles in saline. These hearts were implanted subcutaneously in anti-Gal-producing GT-KO mice without connection to the blood circulation of the recipients. Saline-injected hearts explanted after 2 weeks displayed characteristic necrotic features including extensive infiltration of neutrophils and acidophilic dead cardiomyocytes with no nuclei and no macrophages (Figure 10A). In contrast, hearts injected with α-gal nanoparticles displayed extensive infiltration of macrophages into the injected regions and no neutrophils, despite the absence of any vascular connection to the recipients’ circulation (Figure 10B), [16].

The α-gal nanoparticle-injected hearts explanted after 4 weeks also displayed macrophage migration into areas that were not injected, as well as a conservation of the cardiomyocyte structure and intercalated discs, despite the absence of nuclei (Figure 10C), and in regions with no infiltrating macrophages (Figure 10D). The total size of the dead hearts at 4 weeks post-implantation decreased by 50–80%, whereas the hearts injected with saline completely disappeared within that period. These observations suggest that some of the cytokines secreted by the recruited pro-regenerative macrophages suppress the necrotizing activity of neutrophils, thereby conserving for weeks the structure of dead tissues despite a lack of blood supply to the cardiomyocytes and the absence of nuclei in these cells. The significance of this activity in the observed α-gal nanoparticle regenerative processes awaits further elucidation.

### 5.5. Regeneration of Injured Spinal Cord

Spinal-cord injury is a third injury in which preliminary data suggest that α-gal epitopes can induce regeneration, instead of scar formation. In injuries of the spinal cord, axons of nerve cells are transected. Regeneration of the injured nerves occurs if sprouts developing at the severed end of the axon grow across the lesion gap, “find” distal axonal tubes and grow into them. Such a regenerative process is very limited, as can be inferred from the poor prognosis following spinal cord injuries. In a large proportion of such injuries the damage is irreversible because the default fibrosis of the injured area prevents axonal sprouts from bridging the lesion and connecting with the distal section of the injured axons. Thus, it was of interest to determine whether the injection of α-gal nanoparticles into injured spinal cord lesions can accelerate axonal sprout growth across the lesion and into distal axonal tubes, prior to the occurrence of fibrosis, which prevents this growth.

**Figure 11:**
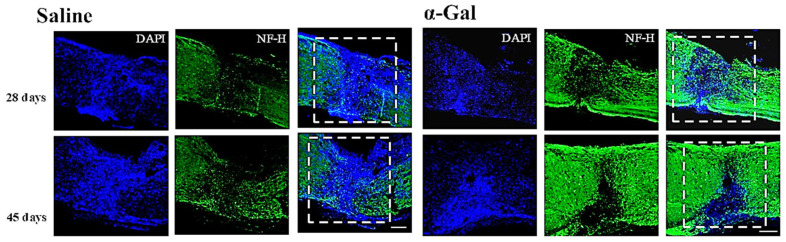
The regenerative effect of α-gal nanoparticles on spinal cord injuries. The treated lesions were viewed as longitudinal sections of specimen obtained 28 and 45 days post-injury and stained with neurofilament specific antibody (NF-H, green) and with DAPI staining nuclei (blue). The dashed square identifies the lesion site. Representative mouse out of 3–4 mice at each time point. Reproduced from Ref. [123] with permission.

Anesthetized anti-Gal-producing GT-KO mice underwent laminectomy at the T9-T10 thoracic segment, and their spinal cord was crushed for 15 s. Subsequently, the injured areas were injected with either 0.5 μL saline, or 0.5 μL α-gal nanoparticles (10 mg/mL in saline). The wound site was sutured, and the mice were monitored for up to 2 months. Histological evaluation at various days post-treatment demonstrated in the α-gal nanoparticle-treated spinal cord a recruitment of macrophages into the treated lesions, which was accompanied by the prevention of fibrosis and increased neo-vascularization due to high VEGF secretion activity by the recruited macrophages [123]. Moreover, the axonal sprouts displayed increased growth, and in some areas, they bridged the lesion to connect with the distal section of the injured axons (Figure 11). The macrophages recruited by the α-gal nanoparticles displayed the M2 macrophage markers of arginase-1 and CD-206. The injected regions displayed neo-vascularization indicated by increased staining of CD-31 on endothelial cells [123]. In addition, it is possible that the prevention of neutrophil infiltration into the lesion site by the recruited macrophages (as demonstrated above) assisted in bridging the lesion by axonal sprouts. No such regenerative changes were observed in saline-treated injured spinal cords. An analysis of sensorimotor parameters in mice demonstrated an improvement in the α-gal nanoparticle-treated mice in locomotor activity in comparison with the control group [123]. Parallel in vitro studies on the interaction between the human microglia cell line HMC3 (macrophages of the nerve system) and anti-Gal-coated α-gal nanoparticles demonstrated an activation of these cells, as indicated by their enlargement and the display of M2 macrophage polarization [124]. These preliminary in vitro findings and the in vivo findings in mice with injured spinal cords suggest that α-gal nanoparticle treatment may be of significance in the restoration of axonal intactness in neurological injuries.

## 6. Limitations

Several clinical trials in cancer patients have reported that in situ exposure of the human immune system to α-gal epitopes results in no significant toxic or other adverse effects [69,88,90,91]. However, there is one limitation common to all α-gal therapies. It is the α-gal syndrome, which describes an allergy to α-gal epitopes, observed in people allergic to meat (beef, pork and lamb). In the USA, individuals with α-gal syndrome produce the anti-Gal IgE isotype following bites by the tick *Ambliyomma americanum* (the “lone star” tick) [125,126,127,128]. A similar allergic response is observed in Europe in individuals bitten by the tick *Ixodes Ricinus* [129], in Asia by *Haemaphysalis longicornis* [130], and in Australia by *Ixodes holocyclus* [131]. Following the digestion of meat in individuals with α-gal syndrome, α-gal epitopes released from the meat on glycolipids, glycoproteins and proteoglycans bind to anti-Gal IgE on mast cells and basophils. This interaction induces degranulation of these cells, resulting within several hours in an allergic reaction manifested by rash, hives, nausea or vomiting, difficulty breathing, low blood pressure, diarrhea, stomach cramps, and pain. Intravenous administration of therapeutic glycoproteins presenting α-gal epitopes to patients with α-gal syndrome may result in life-threatening anaphylactic shock [125]. Thus, it is important to prevent treatment with α-gal therapies in cancer patients, vaccine recipients or patients treated with α-gal nanoparticles, if they have α-gal syndrome or, when asked, if they respond that they are allergic to meat (not poultry or fish). This allergy may be confirmed prior to treatment with a skin test with α-gal glycolipids or glycoproteins [132] and by the assessment of anti-Gal IgE in the serum [125]. Individuals with α-gal syndrome may be treated with α-gal therapies if their allergic reactions are suppressed prior to the treatment by standard anti-allergic treatments.

## 7. Future Directions

The observations described in this review are preliminary, since the area of α-gal epitopes as a therapeutic agent is in its infancy. The anti-Gal-mediated conversion of tumors into anti-TA vaccines was found to be safe in the few Phase I clinical trials that have been performed. Additional research is required for the optimization of α-gal therapies and the prevention of overlapping with the immunosuppressive effects of chemotherapy and radiation treatments. In addition, determining the efficacy of various OV-GT in experimental models such as mouse KO-GT will be helpful in optimizing this virotherapy. It is also important to confirm the complete lack of infection in normal human tissues and cells by OV-GT, in order to prevent any autoimmune-like phenomena, mediated by the natural anti-Gal antibody binding to α-gal epitopes on infected normal cells. Since enveloped OV-GT may present α-gal epitopes on their envelope, they may be susceptible to anti-Gal mediated destruction in the blood. Thus, methods for eliminating α-gal epitopes on OV-GT (e.g., by treatment with α-galactosidase) may contribute to increased efficacy of OV-GT therapies by preventing destruction of these viruses in the circulation.

Vaccine research may benefit from arming vaccinating enveloped viruses with the *GGTA1* gene. It is expected that inactivated α-gal whole-virus vaccines will display a much higher effectiveness in comparison to the same vaccines lacking α-gal epitopes. Future use of SARS-CoV-2_α-gal_ vaccines may further elicit a protective immune response against COVID-19 variants. The lack of any significant toxicity in previous clinical trials with α-gal therapies in cancer patients suggests that, in the absence of α-gal allergies, there is no apparent risk in the expression of α-gal epitopes on viral vaccines.

Regenerative studies of wounds with α-gal nanoparticles in GT-KO mice and GT-KO pigs suggest that clinical trials are required at this stage of research for determining whether this therapy accelerates healing and may result in the scar-free regeneration of skin in wounds and burns. However, the observed α-gal nanoparticle-mediated regeneration of the post-MI ischemic myocardium and of injured spinal cord in GT-KO mice requires further studies to elucidate the mechanism of these processes in mice. In addition, studies in large experimental anti-Gal-producing animal models such as GT-KO pigs or Old-World monkeys are required prior to determining the safety and efficacy of post-MI treatment in humans. Such studies will also help to determine the time-frame after MI, in which α-gal nanoparticles are effective in inducing myocardial regeneration. These models will further assist in determining the effectiveness and safety of α-gal nanoparticle therapy in various surgical procedures, and in mediating regeneration of injured neurons in the peripheral and central nervous systems.

## 8. Concluding Remarks

The interaction between the α-gal epitope and the natural anti-Gal antibody opens a new discipline of harnessing natural antibodies for clinical therapies, because anti-Gal is produced throughout life in large amounts in all humans who are not severely immunocompromised. Controlled α-gal/anti-Gal interactions result in localized activation of the complement system that can kill cells presenting α-gal epitopes. Complement cleavage chemotactic peptides recruit macrophages into the site of complement activation, followed by robust uptake of anti-Gal-opsonized cells, cell membranes and particulate materials by macrophages and dendritic cells. Research on the α-gal epitope as a potential therapeutic agent has demonstrated its use for in situ conversion of tumor lesions into anti-TA vaccines, amplifying the effectiveness of viral vaccines by anti-Gal-mediated targeting of α-gal vaccines for robust uptake by APCs, and inducing regeneration of injured tissue when α-gal epitopes are introduced on nanoparticles into skin wounds and burns, injured myocardium and injured spinal cord. Additional research will help in bringing the gained experience from bench to bed-side and in finding novel ways for exploiting the α-gal/anti-Gal interaction in various clinical settings.

## Data Availability

The data in this review are available upon request.

## Abbreviations

The following abbreviations are used in this manuscript:

α-gal	carbohydrate antigen with the structure Galα1-3Galβ1-4GlcNAc-R
α1,3GT	α1,3galactosyltransferase
ADCC	antibody-dependent-cell-cytolysis
Ad-GT	adenovirus containing the *GGTA1* gene
CDC	complement-dependent-cytolysis
APCs	antigen-presenting-cells
CR1	complement receptor 1
CTL	cytotoxic T-cells
FcγR	Fcγ receptor
GT-KO	mouse or pig knockout for the α1,3galactosyltransferase gene (*GTTA1*)
HCC	hepatic cell carcinoma
HLA	human leukocyte antigen
LAD	left anterior descending
MI	myocardial infarction
NDV	Newcastle disease virus
NDV-GT	NDV containing the *GGTA1* gene
OV	oncolytic virus
OV-GT	oncolytic virus containing the *GGTA1* gene
PVA	polyvinyl alcohol
TAs	tumor antigens
TLC	thin layer chromatography

## References

[1] Galili U., Clark M.R., Shohet S.B., Buehler J., Macher B.A. (1987). Evolutionary relationship between the anti-Gal antibody and the Galα1-3Gal epitope in primates. Proc. Natl. Acad. Sci. USA.

[2] Galili U., Shohet S.B., Kobrin E., Stults C.L.M., Macher B.A. (1988). Man, apes, and Old-World monkeys differ from other mammals in the expression of α-galactosyl epitopes on nucleated cells. J. Biol. Chem..

[3] Basu M., Basu S. (1973). Enzymatic synthesis of blood group related pentaglycosyl ceramide by an α-galactosyltransferase. J. Biol. Chem..

[4] Blake D.D., Goldstein I.J. (1981). An α-D-galactosyltransferase in Ehrlich ascites tumor cells: Biosynthesis and characterization of a trisaccharide (α-D-galacto(1-3)-*N*-acetyllactosamine). J. Biol. Chem..

[5] Betteridge A., Watkins W.M. (1983). Two α-3-D-galactosyltransferases in rabbit stomach mucosa with different acceptor substrate specificities. Eur. J. Biochem..

[6] Van den Eijnden D.H., Blanken W.M., Winterwerp H., Schiphorst W.E. (1983). Identification and characterization of an UDP-Gal: N-acetyllactosaminide alpha-1,3-D-galactosyltransferase in calf thymus. Eur. J. Biochem..

[7] Galili U., Rachmilewitz E.A., Peleg A., Flechner I. (1984). A unique natural human IgG antibody with anti-α-galactosyl specificity. J. Exp. Med..

[8] Avila J.L., Rojas M., Galili U. (1989). Immunogenic Gal α1—3Gal carbohydrate epitopes are present on pathogenic American Trypanosoma and Leishmania. J. Immunol..

[9] McMorrow I.M., Comrack C.A., Sachs D.H., DerSimonian H. (1997). Heterogeneity of human anti-pig natural antibodies cross-reactive with the Gal(α1,3)Galactose epitope. Transplantation.

[10] Parker W., Lin S.S., Yu P.B., Sood A., Nakamura Y.C., Song A., Platt J.L. (1999). Naturally occurring anti-α-galactosyl antibodies: Relationship to xenoreactive anti-α-galactosyl antibodies. Glycobiology.

[11] Galili U., Macher B.A., Buehler J., Shohet S.B. (1985). Human natural anti-α-galactosyl IgG. II. The specific recognition of (α1,3)-linked galactose residues. J. Exp. Med..

[12] Towbin H., Rosenfelder G., Wieslander J., Avila J.L., Rojas M., Szarfman A., Esser K., Nowack H., Timpl R. (1987). Circulating antibodies to mouse laminin in Chagas disease, American cutaneous leishmaniasis, and normal individuals recognize terminal galactosyl [α1-3]-galactose epitopes. J. Exp. Med..

[13] Teneberg S., Lönnroth I., Torres Lopez J.F., Galili U., Olwegard Halvarsson M., Angstrom J., Angstrom J., Karlsson K.A. (1996). Molecular mimicry in the recognition of glycosphingolipids by Galα3Galβ4GlcNAcβ-binding *Clostridium difficile* toxin A, human natural anti-α-galactosyl IgG and the monoclonal antibody Gal-13: Characterization of a binding-active human glycosphingolipid, non-identical with the animal receptor. Glycobiology.

[14] Galili U., Mandrell R.E., Hamadeh R.M., Shohet S.B., Griffiss J.M. (1988). Interaction between human natural anti-α-galactosyl immunoglobulin G and bacteria of the human flora. Infect. Immun..

[15] Mañez R., Blanco F.J., Díaz I., Centeno A., Lopez-Pelaez E., Hermida M., Davies H.F., Katopodis A. (2001). Removal of bowel aerobic gram-negative bacteria is more effective than immunosuppression with cyclophosphamide and steroids to decrease natural alpha-galactosyl IgG antibodies. Xenotransplantation.

[16] Galili U. (2018). The Natural Anti-Gal Antibody as Foe Turned Friend in Medicine.

[17] Galili U., Buehler J., Shohet S.B., Macher B.A. (1987). The human natural anti-Gal IgG. III. The subtlety of immune tolerance in man as demonstrated by crossreactivity between natural anti-Gal and anti-B antibodies. J. Exp. Med..

[18] McMorrow I.M., Comrack C.A., Nazarey P.P., Sachs D.H., DerSimonian H. (1997). Relationship between ABO blood group and levels of Gal α, 3Galactose-reactive human immunoglobulin G. Transplantation.

[19] Larsen R.D., Rajan V.P., Ruff M., Kukowska-Latallo J., Cummings R.D., Lowe J.B. (1989). Isolation of a cDNA encoding murine UDP galactose: ßD-galactosyl-1,4-N-acetyl-D-glucosaminide α1,3-galactosyltransferase: Expression cloning by gene transfer. Proc. Natl. Acad. Sci. USA.

[20] Joziasse D.H., Shaper J.H., Van den Eijnden D.H., Van Tunen A.H., Shaper N.L. (1989). Bovine α1-3galactosyltransferase: Isolation and characterization of a cDNA clone. Identification of homologous sequences in human genomic DNA. J. Biol. Chem..

[21] Galili U., Swanson K. (1991). Gene sequences suggest inactivation of α1-3 galactosyltransferase in catarrhines after the divergence of apes from monkeys. Proc. Natl. Acad. Sci. USA.

[22] Larsen R.D., Rivera-Marrero C.A., Ernst L.K., Cummings R.D., Lowe J.B. (1990). Frameshift and nonsense mutations in a human genomic sequence homologous to a murine UDP-Gal:β-D-Gal [1,4]-D-GlcNAc α[1,3]-galactosyltransferase cDNA. J. Biol. Chem..

[23] Lantéri M., Giordanengo V., Vidal F., Gaudray P., Lefebvre J.C. (2002). A complete α1,3-galactosyltransferase gene is present in the human genome and partially transcribed. Glycobiology.

[24] Henion T.R., Macher B.A., Anaraki F., Galili U. (1994). Defining the minimal size of catalytically active primate α1,3 galactosyltransferase: Structure-function studies on the recombinant truncated enzyme. Glycobiology.

[25] Galili U. (2023). Paleo-immunology of human anti-carbohydrate antibodies preventing primate extinctions. Immunology.

[26] Galili U. (2013). α1,3Galactosyltransferase knockout pigs produce the natural anti-Gal antibody and simulate the evolutionary appearance of this antibody in primates. Xenotransplantation.

[27] Lai L., Kolber-Simonds D., Park K.W., Cheong H.T., Greenstein J.L., Im G.S., Samuel M., Bonk A., Rieke A., Day B.N. (2002). Production of α-1,3-galactosyltransferase knockout pigs by nuclear transfer cloning. Science.

[28] Phelps C.J., Koike C., Vaught T.D., Boone J., Wells K.D., Chen S.H., Ball S., Specht S.M., Polejaeva I.A., Monahan J.A. (2003). Production of α1,3-galactosyltransferase-deficient pigs. Science.

[29] Dor F.J., Tseng Y.L., Cheng J., Moran K., Sanderson T.M., Lancos C.J., Shimizu A., Yamada K., Awwad M., Sachs D.H. (2004). α1,3-Galactosyltransferase gene-knockout miniature swine produce natural cytotoxic anti-Gal antibodies. Transplantation.

[30] Fang J., Walters A., Hara H., Long C., Yeh P., Ayares D., Cooper D.K., Bianchi J. (2012). Anti-gal antibodies in α1,3-galactosyltransferase gene-knockout pigs. Xenotransplantation.

[31] Geyer R., Geyer H., Stirm S., Hunsmann G., Schneider J., Dabrowski U., Dabrowski J. (1984). Major oligosaccharides in the glycoprotein of Friend murine leukemia virus: Structure elucidation by one- and two-dimensional proton nuclear magnetic resonance and methylation analysis. Biochemistry.

[32] Repik P.M., Strizki M., Galili U. (1994). Differential host dependent expression of α-galactosyl epitopes on viral glycoproteins: A study of Eastern equine encephalitis virus as a model. J. Gen. Virol..

[33] Pipperger L., Koske I., Wild N., Müllauer B., Krenn D., Stoiber H., Wollmann G., Kimpel J., von Laer D., Bánki Z. (2019). Xenoantigen-dependent complement-mediated neutralization of LCMV glycoprotein pseudotyped VSV in human serum. J. Virol..

[34] Takeuchi Y., Porter C.D., Strahan K.M., Preece A.F., Gustafsson K., Cosset F.L., Weiss R.A., Collins M.K. (1996). Sensitization of cells and retroviruses to human serum by [α1-3] galactosyltransferase. Nature.

[35] Preece A.F., Strahan K.M., Devitt J., Yamamoto F., Gustafsson K. (2002). Expression of ABO or related antigenic carbohydrates on viral envelopes leads to neutralization in the presence of serum containing specific natural antibodies and complement. Blood.

[36] Welsh R.M., O’Donnell C.L., Reed D.J., Rother R.P. (1998). Evaluation of the Galα1-3Gal epitope as a host modification factor eliciting natural humoral immunity to enveloped viruses. J. Virol..

[37] Rother R.P., Fodor W.L., Springhorn J.P., Birks C.W., Setter E., Sandrin M.S., Squinto S.P., Rollins S.A. (1995). A novel mechanism of retrovirus inactivation in human serum mediated by anti-α-galactosyl natural antibody. J. Exp. Med..

[38] Kim N.Y., Jung W.W., Oh Y.K., Chun T., Park H.Y., Lee H.T., Han I.K., Yang J.M., Kim Y.B. (2007). Natural protection from zoonosis by α-gal epitopes on virus particles in xenotransmission. Xenotransplantation.

[39] Galili U. (2020). Human Natural Antibodies to Mammalian Carbohydrate Antigens as Unsung Heroes Protecting against Past, Present, and Future Viral Infections. Antibodies.

[40] Thall A.D., Maly P., Lowe J.B. (1995). Oocyte Galα,3Gal epitopes implicated in sperm adhesion to the zona pellucida glycoprotein ZP3 are not required for fertilization in the mouse. J. Biol. Chem..

[41] LaTemple D.C., Galili U. (1998). Adult and neonatal anti-Gal response in knock-out mice for α1,3galactosyltransferase. Xenotransplantation.

[42] Tanemura M., Yin D., Chong A.S., Galili U. (2000). Differential immune responses to alpha-gal epitopes on xenografts and allografts: Implications for accommodation in xenotransplantation. J. Clin. Investig..

[43] Cretin N., Bracy J., Hanson K., Iacomini J. (2002). The role of T cell help in the production of antibodies specific for Gal α1-3Gal. J. Immunol..

[44] Benatuil L., Kaye J., Rich R.F., Fishman J.A., Green W.R., Iacomini J. (2005). The influence of natural antibody specificity on antigen immunogenicity. Eur. J. Immunol..

[45] Good A.H., Cooper D.C.K., Malcolm A.J., Ippolito R.M., Koren E., Neethling F.A., Ye Y., Zuhdi N., Lamontagne L.R. (1992). Identification of carbohydrate structures which bind human anti-porcine antibodies: Implication for discordant xenografting in man. Transplant. Proc..

[46] Cooper D.K.C., Good A.H., Koren E., Oriol R., Malcolm A.J., Ippolito R.M., Neethling F.A., Ye Y., Romano E., Zuhdi N. (1993). Identification of α-galactosyl and other carbohydrate epitopes that are bound by human anti-pig antibodies: Relevance to discordant xenografting in man. Transpl. Immunol..

[47] Collins B.H., Cotterell A.H., McCurry K.R., Alvarado C.G., Magee J.C., Parker W., Platt J.L. (1995). Cardiac xenografts between primate species provide evidence for the importance of the alpha-galactosyl determinant in hyperacute rejection. J. Immunol..

[48] Xu Y., Lorf T., Sablinski T., Gianello P., Bailin M., Monroy R., Kozlowski T., Awwad M., Cooper D.K., Sachs D.H. (1998). Removal of anti-porcine natural antibodies from human and nonhuman primate plasma in vitro and in vivo by a Galα1-3Galβ14Glc-R immunoaffinity column. Transplantation.

[49] Simon P.M., Neethling F.A., Taniguchi S., Goode P.L., Zopf D., Hancock W.W., Cooper D.K. (1998). Intravenous infusion of Gala α1-3Gal oligosaccharides in baboon delays hyperacute rejection of porcine heart xenografts. Transplantation.

[50] Galili U. (1993). Interaction of the natural anti-Gal antibody with α-galactosyl epitopes: A major obstacle for xenotransplantation in humans. Immunol. Today.

[51] Sandrin M.S., Vaughan H.A., Dabkowski P.L., McKenzie I.F.C. (1993). Anti-pig IgM antibodies in human serum react predominantly with Gal (αl-3)Gal epitopes. Proc. Natl. Acad. Sci. USA.

[52] Neethling F.A., Joziasse D., Bovin N., Cooper D.K., Oriol R. (1996). The reducing end of alpha-Gal oligosaccharides contributes to their efficiency in blocking natural antibodies of human and baboon sera. Transpl. Int..

[53] Galili U. (2017). α-Gal Nanoparticles in Wound and Burn Healing Acceleration. Adv. Wound Care.

[54] Manches O., Plumas J., Lui G., Chaperot L., Molens J.P., Sotto J.J., Bensa J.C., Galili U. (2005). Anti-Gal-mediated targeting of human B lymphoma cells to antigen-presenting cells: A potential method for immunotherapy using autologous tumor cells. Haematologica.

[55] LaTemple D.C., Henion T.R., Anaraki F., Galili U. (1996). Synthesis of alpha-galactosyl epitopes by recombinant alpha1,3galactosyl transferase for opsonization of human tumor cell vaccines by anti-galactose. Cancer Res..

[56] Shaw S.M., Middleton J., Wigglesworth K., Charlemagne A., Schulz O., Glossop M.S., Whalen G.F., Old R., Westby M., Pickford C. (2019). AGI-134: A fully synthetic α-Gal glycolipid that converts tumors into in situ autologous vaccines, induces anti-tumor immunity and is synergistic with an anti-PD-1 antibody in mouse melanoma models. Cancer Cell Int..

[57] Mumberg D., Wick M., Schreiber H. (1996). Unique tumor antigens redefined as mutant tumor-specific antigens. Semin. Immunol..

[58] Stratton M.R., Campbell P.J., Futreal P.A. (2009). The cancer genome. Nature.

[59] Nik-Zainal S., Van Loo P., Wedge D.C., Alexandrov L.B., Greenman C.D., Lau K.W., Raine K., Jones D., Marshall J., Ramakrishna M. (2012). The life history of 21 breast cancers. Cell.

[60] Zhang L., Conejo-Garcia J.R., Katsaros D., Gimotty P.A., Massobrio M., Regnani G., Makrigiannakis A., Gray H., Schlienger K., Liebman M.N. (2003). Intratumoral T cells, recurrence, and survival in epithelial ovarian cancer. N. Engl. J. Med..

[61] Galon J., Costes A., Sanchez-Cabo F., Kirilovsky A., Mlecnik B., Lagorce-Pagès C., Tosolini M., Camus M., Berger A., Wind P. (2006). Type, density, and location of immune cells within human colorectal tumors predict clinical outcome. Science.

[62] Zinkernagel R.M., Ehl E., Aichele P., Kundig T., Hengartner H. (1997). Antigen localization regulates immune responses in a dose and time dependent fashion: A geographical view of immune reactivity. Immunol. Rev..

[63] Galili U., LaTemple D.C. (1997). Natural anti-Gal antibody as a universal augmenter of autologous tumor vaccine immunogenicity. Immunol. Today.

[64] LaTemple D.C., Abrams J.T., Zhang S.Y., Galili U. (1999). Increased immunogenicity of tumor vaccines complexed with anti-Gal: Studies in knockout mice for α1,3galactosyltransferase. Cancer Res..

[65] Rossi G.R., Mautino M.R., Unfer R.C., Seregina T.M., Vahanian N., Link C.J. (2005). Effective treatment of preexisting melanoma with whole cell vaccines expressing α(1,3)-galactosyl epitopes. Cancer Res..

[66] Deguchi T., Tanemura M., Miyoshi E., Nagano H., Machida T., Ohmura Y., Kobayashi S., Marubashi S., Eguchi H., Takeda Y. (2010). Increased immunogenicity of tumor-associated antigen, mucin 1, engineered to express α-gal epitopes: A novel approach to immunotherapy in pancreatic cancer. Cancer Res..

[67] Galili U., Wigglesworth K., Abdel-Motal U.M. (2007). Intra-tumoral injection of α-gal glycolipids induces xenograft-like destruction and conversion of lesions into endogenous vaccines. J. Immunol..

[68] Abdel-Motal U.M., Wigglesworth K., Galili U. (2009). Intra-tumoral injection of α-gal glycolipids induces a protective anti-tumor T cell response which overcomes Treg activity. Cancer Immunol. Immunother..

[69] Whalen G.F., Sullivan M., Piperdi B., Wasseff W., Galili U. (2012). Cancer immunotherapy by intra-tumoral injection of α-gal glycolipids. Anticancer Res..

[70] Zhang X., He J., Sha Y. (2025). Research progress and development potential of oncolytic vaccinia virus. Chin. Med. J..

[71] Park J.S., Jang W.S., Lee M.E., Kim J., Oh K., Lee N., Ham W.S. (2025). BAP1 as a predictive biomarker of therapeutic response to oncolytic vaccinia virus for metastatic renal cell carcinoma therapy. Cancer Immunol. Immunother..

[72] Davar D., Carneiro B.A., Dy G.K., Sheth S., Borad M.J., Harrington K.J., Patel S.P., Galanis E., Samson A., Agrawal S. (2024). Phase I study of a recombinant attenuated oncolytic virus, MEDI5395 (NDV-GM-CSF), administered systemically in combination with durvalumab in patients with advanced solid tumors. J. Immunother. Cancer.

[73] Yang H., Tian J., Zhao J., Zhao Y., Zhang G. (2024). The Application of Newcastle Disease Virus (NDV): Vaccine Vectors and Tumor Therapy. Viruses.

[74] Betancourt D., Ramos J.C., Barber G.N. (2015). Retargeting Oncolytic Vesicular Stomatitis Virus to Human T-Cell Lymphotropic Virus Type 1-Associated Adult T-Cell Leukemia. J. Virol..

[75] Daniel L., Ndiaye K.S., Giguère H., Custeau L.J., Léger J.L., St-Cyr G., Ndongo N.E., Richard P.O., Tai L.H. (2025). Intravesical VSVd51-GM-CSF virotherapy is superior to BCG in treating bladder cancer in preclinical and translational models. Mol. Ther. Oncol..

[76] Zhang K.J., Zhang J., Wu Y.M., Qian J., Liu X.J., Yan L.C., Ndongo N.E., Richard P.O., Tai L.H. (2012). Complete eradication of hepatomas using an oncolytic adenovirus containing AFP promoter controlling E1A and an E1B deletion to drive IL-24 expression. Cancer Gene Ther..

[77] Pakola S.A., Ojala N., Kudling T.V., Clubb J.H.A., Jirovec E., van der Heijden M., Arias V., Haybout L., Basnet S., Grönberg-Vähä-Koskela S. (2025). Oncolytic adenovirus encoding variant interleukin-2 combined with chemotherapy enables PD-L1 inhibition in pancreatic cancer models. Cancer Immunol. Immunother..

[78] Shalhout S.Z., Miller D.M., Emerick K.S., Kaufman H.L. (2023). Therapy with oncolytic viruses: Progress and challenges. Nat. Rev. Clin. Oncol..

[79] Musher B.L., Rowinsky E.K., Smaglo B.G., Abidi W., Othman M., Patel K., Jawaid S., Jing J., Brisco A., Leen A.M. (2024). LOAd703 an oncolytic virus-based immunostimulatory gene therapy, combined with chemotherapy for unresectable or metastatic pancreatic cancer (LOKON001): Results from arm 1 of a non-randomised, single-centre, phase 1/2 study. Lancet Oncol..

[80] Du W., Na J., Zhong L., Zhang P. (2025). Advances in preclinical and clinical studies of oncolytic virus combination therapy. Front. Oncol..

[81] Rangsitratkul C., Lawson C., Bernier-Godon F., Niavarani S.R., Boudaud M., Rouleau S., Gladu-Corbin A.O., Surendran A., Ekindi-Ndongo N., Koti M. (2022). Intravesical immunotherapy with a GM-CSF armed oncolytic vesicular stomatitis virus improves outcome in bladder cancer. Mol. Ther. Oncolytics.

[82] Raja J., Ludwig J.M., Gettinger S.N., Schalper K.A., Kim H.S. (2018). Oncolytic virus immunotherapy: Future prospects for oncology. J. Immunother. Cancer.

[83] Shen Y., Bai X., Zhang Q., Liang X., Jin X., Zhao Z., Song W., Tan Q., Zhao R., Jia W. (2025). Oncolytic virus VG161 in refractory hepatocellular carcinoma. Nature.

[84] Russell S.J., Barber G.N. (2018). Oncolytic Viruses as Antigen-Agnostic Cancer Vaccines. Cancer Cell.

[85] Deriy L., Chen Z., Gao G., Galili U. (2002). Expression of α-Gal epitopes (Galα1-3Gal β1-4GlcNAc-R) on human cells following transduction with adenovirus vector containing α1,3galactosyltransferase cDNA. Glycobiology.

[86] Deriy L., Ogawa H., Gao G., Galili U. (2005). In vivo targeting of vaccinating tumor cells to antigen presenting cells by a gene therapy method with adenovirus containing the alpha1,3galactosyltransferase gene. Cancer Gene Ther..

[87] Galili U., Zhao Y. (2026). Potential induction of protective anti-tumor immune response in cancer patients by oncolytic viruses containing the GGTA1 gene. Eur. J. Cancer.

[88] Zhong L., Gan L., Wang B., Wu T., Yao F., Gong W., Peng H., Deng Z., Xiao G., Liu X. (2025). Hyperacute rejection-engineered oncolytic virus for interventional clinical trial in refractory cancer patients. Cell.

[89] Galili U. (2004). Autologous tumor vaccines processed to express α-gal epitopes: A practical approach to immunotherapy in cancer. Cancer Immunol. Immunother..

[90] Qiu Y., Xu M.B., Yun M.M., Wang Y.Z., Zhang R.M., Ou-Yang X.H., Yun S. (2011). Hepatocellular Carcinoma-specific Immunotherapy with Synthesized α1,3 Galactosyl Epitope-Pulsed Dendritic-cells and Cytokine-Induced Killer Cells. World J. Gastroenterol..

[91] Qiu Y., Xu M.B., Yun M.M., Dong X., Xu M., Zhou E., Yun F., Su W., Liu C., Zhao H. (2016). Combination of Cytokine-Induced Killer and Dendritic-cells Pulsed with Antigenic α-1,3 galactosyl Epitope-Enhanced Lymphoma Cell Membrane for Effective B-Cell Lymphoma Immunotherapy. Cytotherapy.

[92] Galili U., Repik P.M., Anaraki F., Mozdzanowska K., Washko G., Gerhard W. (1996). Enhancement of antigen presentation of influenza virus hemagglutinin by the natural human anti-Gal antibody. Vaccine.

[93] Henion T.R., Gerhard W., Anaraki F., Galili U. (1997). Synthesis of α-gal epitopes on influenza virus vaccines, by recombinant α1,3galactosyltransferase, enables the formation of immune complexes with the natural anti-Gal antibody. Vaccine.

[94] Abdel-Motal U.M., Guay H.M., Wigglesworth K., Welsh R.M., Galili U. (2007). Immunogenicity of influenza virus vaccine is increased by anti-Gal-mediated targeting to antigen-presenting cells. J. Virol..

[95] Yan L.M., Lau S.P.N., Poh C.M., Chan V.S.F., Chan M.C.W., Peiris M., Poon L.L.M. (2020). Heterosubtypic Protection Induced by a Live Attenuated Influenza Virus Vaccine Expressing Galactose-α-1,3-Galactose Epitopes in Infected Cells. mBio.

[96] Abdel-Motal U., Wang S., Lu S., Wigglesworth K., Galili U. (2006). Increased immunogenicity of human immunodeficiency virus gp120 engineered to express Galα1-3Galβ1-4GlcNAc-R epitopes. J. Virol..

[97] Abdel-Motal U.M., Wang S., Awwad S., Lu S., Wigglesworth K., Galili U. (2010). Increased immunogenicity of HIV-1 p24 and gp120 following immunization with gp120/p24 fusion protein vaccine expressing α-gal epitopes. Vaccine.

[98] Galili U. (2020). Amplifying immunogenicity of prospective COVID-19 vaccines by glycoengineering the coronavirus glycan-shield to present α-gal epitopes. Vaccine.

[99] Galili U. (2021). COVID-19 variants as moving targets and how to stop them by glycoengineered whole-virus vaccines. Virulence.

[100] Azeem M., Cancemi P., Mukhtar F., Marino S., Peri E., Di Prima G., De Caro V. (2025). Efficacy and limitations of SARS-CoV-2 vaccines—A systematic review. Life Sci..

[101] Watanabe Y., Allen J.D., Wrapp D., McLellan J.S., Crispin M. (2020). Site-specific glycan analysis of the SARS-CoV-2 spike. Science.

[102] Smith D.F., Larsen R.D., Mattox S., Lowe J.B., Cummings R.D. (1990). Transfer and expression of a murine UDP-Gal:b-D-Gal-α1,3-galactosyltransferase gene in transfected Chinese hamster ovary cells. Competition reactions between the α1,3-galactosyltransferase and the endogenous α 2,3-sialyltransferase. J. Biol. Chem..

[103] Porrello E.R., Mahmoud A.I., Simpson E., Hill J.A., Richardson J.A., Olson E.N., Sadek H.A. (2011). Transient regenerative potential of the neonatal mouse heart. Science.

[104] Haubner B.J., Adamowicz-Brice M., Khadayate S., Tiefenthaler V., Metzler B., Aitman T., Penninger J.M. (2012). Complete cardiac regeneration in a mouse model of myocardial infarction. Aging.

[105] Godwin J.W., Debuque R., Salimova E., Rosenthal N.A. (2017). Heart regeneration in the salamander relies on macrophage-mediated control of fibroblast activation and the extracellular landscape. NPJ Regen. Med..

[106] Ye L., D’Agostino G., Loo S.J., Wang C.X., Su L.P., Tan S.H., Tee G.Z., Pua C.J., Pena E.M., Cheng R.B. (2018). Early Regenerative Capacity in the Porcine Heart. Circulation.

[107] Zhu W., Zhang E., Zhao M., Chong Z., Fan C., Tang Y., Hunter J.D., Borovjagin A.V., Walcott G.P., Chen J.Y. (2018). Regenerative potential of neonatal porcine hearts. Circulation.

[108] Aurora A.B., Porrello E.R., Tan W., Mahmoud A.I., Hill J.A., Bassel-Duby R., Sadek H.A., Olson E.N. (2014). Macrophages are required for neonatal heart regeneration. J. Clin. Investig..

[109] Lavine K.J., Epelman S., Uchida K., Weber K.J., Nichols C.G., Schilling J.D., Ornitz D.M., Randolph G.J., Mann D.L. (2014). Distinct macrophage lineages contribute to disparate patterns of cardiac recovery and remodeling in the neonatal and adult heart. Proc. Natl. Acad. Sci. USA.

[110] Natarajan N., Abbas Y., Bryant D.M., Gonzalez-Rosa J.M., Sharpe M., Uygur A., Cocco-Delgado L.H., Ho N.N., Gerard N.P., Gerard C.J. (2018). Complement Receptor C5aR1 Plays an Evolutionarily Conserved Role in Successful Cardiac Regeneration. Circulation.

[111] Nahrendorf M., Swirski F.K., Aikawa E.L., Wurdinger T., Figueiredo J.S., Libby P., Weissleder R., Pittet M.J. (2007). The healing myocardium sequentially mobilizes two monocyte subsets with divergent and complementary functions. J. Exp. Med..

[112] Prabhu S.D., Frangogiannis N.G. (2016). The Biological Basis for Cardiac Repair After Myocardial Infarction: From Inflammation to Fibrosis. Circ. Res..

[113] Mahmoud A.I., Porrello E.R. (2012). Turning back the cardiac regenerative clock: Lessons from the neonate. Trends Cardiovasc. Med..

[114] Galili U., Wigglesworth K., Abdel-Motal U.M. (2010). Accelerated healing of skin burns by anti-Gal/alpha-gal liposomes interaction. Burns.

[115] Wigglesworth K.M., Raski W.J., Mishra R., Szomolanyi-Tsuda E., Greiner D.L., Galili U. (2011). Rapid recruitment and activation of macrophages by anti-Gal/α-Gal liposome interaction accelerates wound healing. J. Immunol..

[116] Galili U., Zhu Z., Chen J., Goldufsky J.W., Schaer G.L. (2021). Near Complete Repair after Myocardial Infarction in Adult Mice by Altering the Inflammatory Response with Intramyocardial Injection of α-Gal Nanoparticles. Front. Cardiovasc. Med..

[117] Galili U., Goldufsky J.W., Schaer G.L. (2022). α-Gal Nanoparticles Mediated Homing of Endogenous Stem Cells for Repair and Regeneration of External and Internal Injuries by Localized Complement Activation and Macrophage Recruitment. Int. J. Mol. Sci..

[118] Galili U., Li J., Schaer G.L. (2024). Regeneration in Mice of Injured Skin, Heart, and Spinal Cord by α-Gal Nanoparticles Recapitulates Regeneration in Amphibians. Nanomaterials.

[119] Samadi A., Buro J., Dong X., Weinstein A., Lara D.O., Celie K.B., Wright M.A., Gadijko M.A., Galili U., Spector J.A. (2022). Topical α-Gal Nanoparticles Enhance Wound Healing in Radiated Skin. Ski. Pharmacol. Physiol..

[120] Kaymakcalan O.E., Karinja S., Abadeer A., Dong X., Jin J.L., Galili U., Spector J.A. (2018). Antigen-Mediated, Macrophage-Stimulated, Accelerated Wound Healing Using α-Gal Nanoparticles. Ann. Plast. Surg..

[121] Kaymakcalan O.E., Abadeer A., Goldufsky J.W., Galili U., Karinja S.J., Dong X., Jin J.L., Samadi A., Spector J.A. (2020). Topical α-gal nanoparticles accelerate diabetic wound healing. Exp. Dermatol..

[122] Hurwitz Z.M., Ignotz R., Lalikos J.F., Galili U. (2012). Accelerated porcine wound healing after treatment with α-gal nanoparticles. Plast. Reconstr. Surg..

[123] Gopalakrishnan B., Galili U., Saenger M., Burket N.J., Koss W., Lokender M.S., Wolfe K.M., Husak S.J., Stark C.J., Solorio L. (2024). α-Gal Nanoparticles in CNS Trauma: II. Immunomodulation Following Spinal Cord Injury (SCI) Improves Functional Outcomes. Tissue Eng. Regen. Med..

[124] Gopalakrishnan B., Galili U., Dunbar A., Solorio L., Shi R., Li J. (2024). alpha-Gal Nanoparticles in CNS Trauma: I. In Vitro Activation of Microglia Towards a Pro-Healing State. Tissue Eng. Regen. Med..

[125] Chung C.H., Mirakhur B., Chan E., Le Q.T., Berlin J., Morse M., Murphy B.A., Satinover S.M., Hosen J., Mauro D. (2008). Cetuximab-induced anaphylaxis and IgE specific for galactose-α-1,3-galactose. N. Engl. J. Med..

[126] Commins S.P., James H.R., Kelly L.A., Pochan S.L., Workman L.J., Perzanowski M.S., Kocan K.M., Fahy J.V., Nganga L.W., Ronmark E. (2011). The relevance of tick bites to the production of IgE antibodies to the mammalian oligosaccharide galactose-α-1,3-galactose. J. Allergy Clin. Immunol..

[127] Platts-Mills A.E., Schuyler A.J., Hoyt A.E., Commins S.P. (2015). Delayed anaphylaxis involving IgE to galactose-α 1,3galactose. Curr. Allergy Asthma Rep..

[128] Wilson J.M., Erickson L., Levin M., Ailsworth S.M., Commins S.P., Platts-Mills T.A.E. (2024). Tick bites, IgE to galactose-alpha-1,3-galactose and urticarial or anaphylactic reactions to mammalian meat: The alpha-gal syndrome. Allergy.

[129] Hamsten C., Starkhammar M., Tran T.A., Johansson M., Bengtsson U., Ahlén G., Sällberg M., Grönlund H., van Hage M. (2013). Identification of galactose-α-1,3-galactose in the gastrointestinal tract of the tick *Ixodes ricinus*; possible relationship with red meat allergy. Allergy.

[130] Chinuki Y., Ishiwata K., Yamaji K., Takahashi H., Morita E. (2016). *Haemaphysalis longicornis* tick bites are a possible cause of red meat allergy in Japan. Allergy.

[131] Van Nunen S.A., O’Connor K.S., Clarke L.R., Boyle R.X., Fernando S.L. (2009). An association tick bite reactions and red meat allergy in humans. Med. J. Aust..

[132] Albertini M.R., Ranheim E.A., Zuleger C.L., Sondel P.M., Hank J.A., Bridges A., Newton M.A., McFarland T., Collins J., Clements E. (2016). Phase I study to evaluate toxicity and feasibility of intratumoral injection of α-gal glycolipids in patients with advanced melanoma. Cancer Immunol. Immunother..

